# An Efficient Synthesis of Aldohexose-Derived Piperidine Nitrones: Precursors of Piperidine Iminosugars

**DOI:** 10.3390/molecules18056021

**Published:** 2013-05-21

**Authors:** Hui Zhao, Wen-Bo Zhao, Jian-She Zhu, Yue-Mei Jia, Chu-Yi Yu

**Affiliations:** Beijing National Laboratory for Molecular Science, CAS Key Laboratory of Molecular Recoganition and Function, Institute of Chemistry, Chinese Academy of Sciences, Beijing 100190, China

**Keywords:** aldohexose-derived cyclic nitrones, synthesis, piperidine iminosugars

## Abstract

d-Glucopyranose-derived and l-idopyranose-derived piperidine nitrones were synthesized in good overall yields through six-step reaction sequence starting from readily available 2,3,4,6-tetra-*O*-benzyl-d-glucopyranose. The method is efficient and could be general for the synthesis of aldohexose-derived piperidine nitrones which are precursors of piperidine iminosugars.

## 1. Introduction

**Figure 1:**
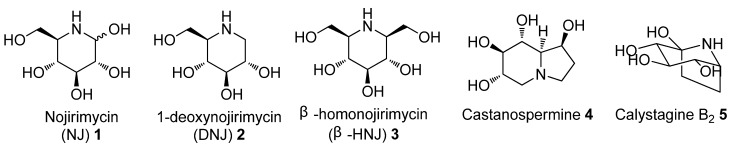
Some naturally occurring azapyranose alkaloids.

Because of their remarkable biological activities [1,2,3], iminosugars, the ‘nitrogen-in-the-ring’ analogues of pyranoses and furanoses, have attracted considerable attention from synthetic organic chemists [4,5,6,7]. Over the last 40 years, a very large number of azapyranose sugars, * i.e.*, piperidine iminosugars, have been isolated from various plants and animal sources (Figure 1). Nearly all these naturally-occurring iminosugars, as well as the majority of synthetic azapyranose sugars, elicit some sort of biological response [6]. Although great efforts have been made and numerous synthetic procedures have been developed for the synthesis of iminosugars [8,9,10,11,12,13,14,15,16,17,18,19,20,21,22], efficient methods for the rapid generation of azapyranoses with the structural diversity necessary for in-depth structure-activity studies are still lacking. Due to the versatile reactivities of the nitrone functionality, synthetic methods based on sugar-derived cyclic nitrones have emerged as a potentially powerful strategy for the diversity-oriented synthesis of iminosugars among the existing approaches [23,24,25,26,27,28,29,30,31,32,33].

As a result of their capability of undergoing a variety of synthetically useful reactions, such as 1,3-dipolar cycloadditions [34,35,36,37,38,39,40], nucleophilic additions [39,41,42,43,44,45,46,47,48], and pinacol-type coupling reactions [49,50,51,52,53,54,55,56], *etc.*, nitrones [57,58] have been shown to be powerful synthetic intermediates for the construction of structurally complex molecules [59,60,61]. Polyhydroxylated cyclic nitrones or sugar-derived cyclic nitrones, which have been widely used in the synthesis of various natural and biologically active nitrogen-containing compounds [27,28,32,33,62,63,64,65], are especially valuable in organic synthesis. The piperidine nitrones, may serve as the powerful synthons or precursors for the synthesis of naturally-occurring piperidine iminosugars, especially for the diversity-oriented synthesis.

**Scheme 1:**
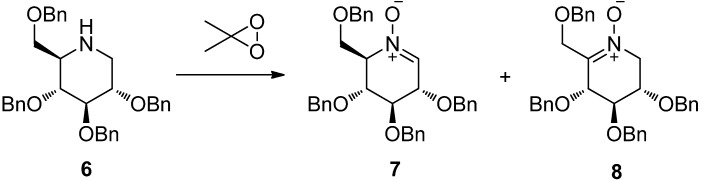
van den Broek’s synthesis of pyranose-derived nitrone.

Although, methods for the synthesis of cyclic nitrones have been well documented [66], including oxidation of hydroxylamine [67,68,69], amines [70,71,72,73], and imines [74,75], condensation of ketones with hydroxylamines [76,77], and *N*-alkylation of oximes [78,79,80], efficient synthetic methods for the preparation of pyranose-derived nitrones are still lacking. Several syntheses of pentopyranose-derived piperidine nitrones have been reported before [81,82,83], however, to the best of our knowledge, the only synthesis of a hexopyranose-derived piperidine nitrone so far has been reported in 1996 by van den Broek and co-workers [84] who described the preparation of pyranose-derived nitrones by oxidation of 2,3,4,6-*O*-benzyl-DNJ **6** employing 2,2-dimethydioxirane (DMDO) as oxidizing agent (Scheme 1). This method, however, has obvious drawbacks in that the regioselectivity of the oxidation was poor and the oxidation of the piperidine resulted in the formation of an inseparable mixture of two regio-isomers,* i.e.*, the aldo and keto nitrones **7** and **8**.

In the context of our ongoing projects on the synthesis and application of sugar-derived cyclic nitrones [80], herein we report an efficient synthesis of d-glucopyranose- and l-idopyranose-derived piperidine nitrones. We intend to develop a general and efficient method for the synthesis of aldohexose-derived piperidine nitrones, which could be powerful synthons for the synthesis of iminosugars containing a piperidine moiety in the molecules.

## 2. Results and Discussion

### 2.1. The Unsuccessful N-alkylation Strategy

**Scheme 2:**
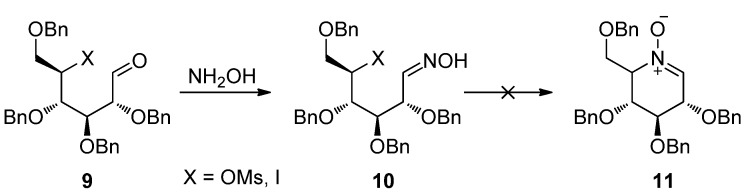
Unsuccessful intramolecular *N*-alkylation strategy.

Intramolecular *N*-alkylation, the most widely used strategy in cyclic nitrone synthesis, was not applicable here. Oxime **10**, prepared from d-glucose-derived aldehyde **9**, failed under various conditions to undergo the predicted intramolecular *N*-alkylation to give the desired hexopyranose-derived piperidine nitrone **11** (Scheme 2). It seems that intramolecular nucleophilic substitution to form hexopyranose-type piperidine rings is less favorable than for furanose- or pentopyranose rings, possibly due to the relatively poor reactivity of the leaving group at C5 of the hexopyranose.

In order to avoid the low regioselectivity and ring formation problems of the method of oxidation and intramolecular *N*-alkylation, we turned to a less employed strategy in sugar-derived cyclic nitrone synthesis—the intramolecular condensation of aldehydes with hydroxylamines [76,77,85,86]—which has been successfully applied in the synthesis of aldohexose-derived azepane nitrones.

### 2.2. The Preparation of Diethyl Dithioacetal

The readily available 2,3,4,6-tetra-*O*-benzyl-d-glucopyranose (**12**) was chosen as the starting material for our investigation on the synthesis of aldohexose-derived piperidine nitrones, which is expected to give two nitrones,* i.e.*, a d-glucopyranose-derived nitrone and an l-idopyranose-derived nitrone.

**Table 1:** Screening of conditions of preparing diethyl dithioacetal. 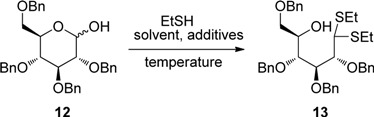

Entry	Additives	Eq.	Solvent	Tem. (°C)	Yield ^a^ (%)
1	TMSCl	0.2	CHCl_3_	RT	-
2	BF_3_·Et_2_O	0.2	CHCl_3_	RT	-
3	AcCl	0.2	CH_2_Cl_2_	RT	-
5	conc. HCl	excess	MeOH	RT	-
6	HCl (g)	excess	Dioxane	RT	56
7	BDMS	0.2	CH_3_CN	0- RT	trace
8	BDMS	0.2	CH_2_Cl_2_	0- RT	trace
9	BDMS	0.2	EtSH	RT	84 ^b^

^a^ Isolated yield. ^b^ Reference [82].

First of all, in order to find the optimal reaction conditions for the synthesis of the key intermediate, the alcohol **13**, through the pyranose hemiacetal ring opening by ethanethiol, various reagents and reaction conditions has been screened (Table 1). It turned out that the method using bromodimethylsulfonium bromide (BDMS), reported by Khan’s group [87], gave the best result.

### 2.3. Completion of the Synthesis

**Scheme 3:**
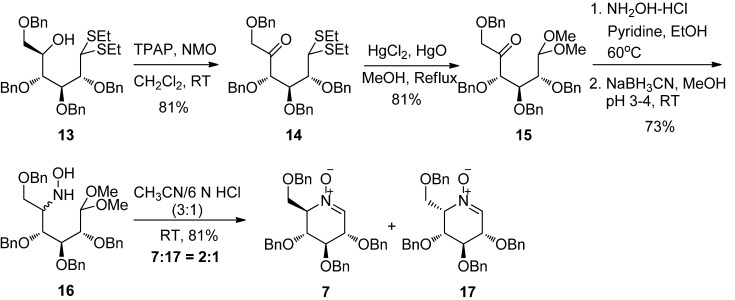
Completion of the synthesis of d-glucopyranose-derived and l-idopyranose-derived piperidine nitrones.

Oxidation of the resulting alcohol **13** using tetrapropylammonium perruthenate (TPAP) [88] gave the corresponding ketone **14 **in 81% yield (Scheme 3). Other methods of oxidation, such as the Swern oxidation, Corey-Suggs oxidation (PCC), and Dess-Martin oxidation (DMP), all failed to oxidize **13** into the desired ketone **14**. To transform the diethyl dithiolacetal protecting group of **14** into more acid-labile dimethyl acetal protecting group, the ketone **14** was treated with mercury salts in the presence of methanol to form the dimethyl acetal protected ketone **15** in 81% yield. 

Then, condensation of ketone **15** with NH_2_OH, followed by reduction of the resulting oxime with sodium cyanoborohydride gave the hydroxylamine** 16** as an inseparable mixture of two C5 isomers in 73% yield. A pure analytical sample of the major C5 isomer of **16** was obtained by flash column chromatography and its structure was determined by NMR (see Supporting Information). Finally, acidic hydrolysis of the dimethyl acetal **16** by CH_3_CN/6 N HCl and subsequent intramolecular condensation of the hydroxylamine and aldehyde produced a mixture of two products, the d-gluco-pyranose-derived piperidine nitrone **7** and l-idopyranose-derived piperidine nitrone **17**, in 81% total yield, which are easily separated by column chromatography on silica gel with a ratio of 2:1. The structures of the two nitrones have been determined undoubtedly by NMR and NOESY experiments (see Supplementary Materials). It is worth noting that the d-glucopyranose-derived piperidine nitrone **7** was found to be quite stable, even at room temperature, rather than unstable as has been reported before [84], while the l-idopyranose-derived piperidine nitrone **17** is less stable, and gradually decomposed at room temperature, although it was stable at low temperature when stored in a fridge.

## 3. Experimental

### 3.1. General Methods

All reagents were used as received from commercial sources without further purification or prepared as described in the literature. Reactions were stirred using Teflon-coated magnetic stirring bars. Analytical TLC was performed with 0.20 mm silica gel 60F plates with 254 nm fluorescent indicator. TLC plates were visualized by ultraviolet light or by treatment with a spray of Pancaldi reagent [(NH_4_)_6_MoO_4_, Ce(SO_4_)_2_, H_2_SO_4_, H_2_O]. Chromatographic purification of products was carried out by flash column chromatography on silica gel (200–300 mesh). Infrared spectra were recorded on a JASCO FT/IR-480 plus Fourier transform spectrometer. NMR spectra were measured in CDCl_3_ (with TMS as internal standard) on a Bruker AV300 (^1^H at 300 MHz, ^13^C at 75 MHz) magnetic resonance spectrometer. Chemical shifts (δ) are reported in ppm, and coupling constants (*J*) are in Hz. The following abbreviations were used to explain the multiplicities: s = singlet, d = doublet, t = triplet, q = quartet, m = multiplet. High-resolution mass spectra (HRMS) were recorded on a Thermo Scientific LTQ/FT mass spectrometer or a GCT mass spectrometer. Polarimetry was carried out using an Optical Activity AA-10R polarimeter and the measurements were made at the sodium D-line with a 0.5 dm pathlength cell. Concentrations (c) are given in gram per 100 mL.

### 3.2. Synthesis

*(2R,3R,4S,5R)-1,3,4,5-Tetrakis(benzyloxy)-6,6-bis(ethylthio)hexan-2-ol* (**13**). A catalytic amount (7 mg, 0.03 mmol) of bromodimethylsulfonium bromide (BDMS) was added to a stirred mixture of 2,3,4,6-tetra-*O*-benzyl-d-glucopyranose (**12**, 500 mg, 1 mmol) and ethanethiol (5 mL) at room temperature. The mixture was stirred for 2 h, and the mixture gradually became clear. TLC indicated the disappearance of starting material, then the excess ethanethiol was removed *in vacuo*. The residual products were dissolved in ethyl acetate, and washed with aq. NaHCO_3_ (3 × 10 mL). Removal of the solvent *in vacuo* after dried over MgSO_4_, the crude product was purified by column chromatography on silica gel (petroleum ether-ethyl acetate = 40:1–20:1) to give compound **13** (552 mg, 84%) as a colourless oil. ^1^H-NMR (CDCl_3_) δ 7.48–7.25 (m, 20H), 4.95 (d, *J* = 11.1 Hz, 1H), 4.86 (d, *J* = 11.2 Hz, 1H), 4.85 (d, *J* = 11.2 Hz, 1H), 4.73 (d, *J* = 11.2 Hz, 1H), 4.64–4.54 (m, 4H), 4.33 (dd, *J* = 6.6, 3.6 Hz, 1H), 4.20 (dd, *J* = 6.6, 3.7 Hz, 1H), 4.13 (dt, *J* = 10.1, 4.9 Hz, 1H), 3.98 (d, *J* = 3.7 Hz, 1H), 3.77 (dd, *J* = 7.0, 3.6 Hz, 1H), 3.71 (d, *J* = 3.8 Hz, 1H), 3.69 (d, *J* = 5.5 Hz, 1H), 3.15 (d, *J* = 4.7 Hz, 1H), 2.72 (q, *J* = 7.4 Hz, 2H), 2.67–2.53 (m, 2H), 1.23 (t,* J* = 7.2 Hz, 3H), 1.22 (t, *J* = 7.6 Hz, 3H). ^13^C-NMR (CDCl_3_) δ 138.5, 138.1, 137.9, 128.5, 128.4, 128.4, 128.3, 128.2, 127.9, 127.9, 127.78, 127.75, 127.5, 82.7, 80.0, 75.3, 74.9, 73.5, 72.8, 71.4, 70.9, 53.8, 25.5, 25.3, 14.5. [lit. [82] ^1^H-NMR (400 MHz; CDCl_3_) δ 7.38–7.23 (m, 20H), 4.89 (d, *J* = 11.2 Hz, 1H), 4.80 (d, *J* = 11.2, 1H), 4.79 (d, , *J* = 11.6, 1H), 4.67 (d, *J* = 11.6 Hz, 1H), 4.59–4.49 (m, 4H), 4.27 (dd, *J* = 6.8, 3.6 Hz, 1H), 4.14 (dd, *J* = 6.8, 4.0 Hz, 1H), 4.15–4.03 (m, 1H), 3.93 (d, *J* = 4.0 Hz, 1H), 3.71 (dd, *J* = 6.8, 3.6 Hz, 1H), 3.66 (dd, *J* = 10.0, 4.0 Hz, 1H), 3.61 (dd, *J* = 10.0, 5.6 Hz, 1H), 3.08 (d, *J* = 5.2 Hz, 1H), 2.66 (q, *J* = 7.6 Hz, 2H), 2.60–2.50 (m, 2H), 1.18 (t, *J* = 7.2 Hz, 3H), 1.17 (t, *J* = 7.6 Hz, 3H); ^13^C-NMR (100 MHz; CDCl_3_) δ 138.7, 138.4, 138.1, 128.6, 128.4, 128.3, 128.2, 128.03, 128.0, 127.96, 127.9, 127.6, 82.9, 80.3, 77.5, 75.5, 75.1, 73.7, 73.0, 71.6, 71.1, 54.0, 25.7, 25.5, 14.6.]

*(3S,4S,5R)-1,3,4,5-Tetrakis(benzyloxy)-6,6-bis(ethylthio)hexan-2-one* (**14**). Solid TPAP (68 mg, 0.19 mmol) was added portionwise to a stirred mixture of dithioacetal **13** (2.49 g, 3.85 mmol), 4-methylmorpholine *N*-oxide (NMO) (676 mg, 5.77 mmol) and powdered 4 Å molecular sieves (2 g) in dry CH_2_Cl_2_ (8 mL) at 0 °C under nitrogen. The mixture was stirred for 45 min at room temperature, filtered through a short pad of silica and eluted with ethyl acetate. The filtrate was concentrated *in vacuo* to give a yellow oil. Column chromatography (petroleum ether-ethyl acetate = 6:1) provided ketone **14** (2.07 g, 81%) as a colourless oil. ^1^H-NMR (CDCl_3_) δ 7.43–7.13 (m, 20H), 4.81 (d, *J* = 10.9 Hz, 1H), 4.73 (d, *J* = 11.2 Hz, 1H), 4.70 (s, 1H), 4.66 (s, 1H), 4.51 (d, *J* = 11.2 Hz, 1H), 4.41–4.32 (m, 4H), 4.23 (d, *J* = 17.5 Hz, 1H), 4.16 (d, *J* = 4.2 Hz, 1H), 4.11 (d, *J* = 17.6 Hz, 1H), 4.03 (dd, *J* = 5.8, 4.6 Hz, 1H), 3.75 (d, *J* = 4.4 Hz, 1H), 2.69–2.60 (m, 2H), 2.55 (dd, *J* = 14.9, 7.8 Hz, 2H), 1.18 (t, *J* = 7.2 Hz, 3H,), 1.17 (t, *J* = 7.6 Hz, 3H). ^13^C-NMR (CDCl_3_) δ 206.5, 138.3, 137.8, 137.4, 136.9, 128.6, 128.43, 128.38, 128.35, 128.24, 128.21, 128.0, 127.9, 127.8, 127.5, 81.7, 81.4, 80.8, 75.2, 75.1, 74.4, 73.4, 73.3, 53.4, 25.3, 14.4. [lit. [89] ^1^H-NMR (300 MHz; CDCl_3_) δ 7.43–7.18 (20H, m), 4.82 (d, *J* =11.0 Hz, 1H), 4.74 (d, *J* = 11.0 Hz, 1H), 4.70 (d, *J* = 11.0 Hz, 1H), 4.69 (d, *J* = 11.0 Hz, 1H), 4.52 (d, *J* = 11.0 Hz, 1H), 4.41–4.33 (m, 4H), 4.24 (d, *J* = 18.0 Hz, 1H), 4.16 (d, *J* = 4.0 Hz, 1H), 4.12 (d, *J* = 18.0 Hz, 1H), 4.04 (dd, *J* = 6.0, 4.5 Hz, 1H), 3.75 (d, *J* = 4.5 Hz, 1H), 2.64 (m, 2H), 2.56 (m, 2H), 1.19 (t, *J* = 7.5 Hz, 3H), 1.18 (t, *J* = 7.5 Hz, 3H); ^13^C-NMR (75 MHz; CDCl_3_) δ 206.5, 138.3, 137.7, 137.3, 136.8, 128.6, 128.46, 128.43, 128.38, 128.35, 128.25, 128.21, 127.9, 127.87, 127.85, 127.5, 81.6, 81.4, 80.7, 75.2, 75.1, 74.3, 73.34, 73.31, 53.3, 25.2, 14.4].

*(3S,4R,5R)-1,3,4,5-Tetrakis(benzyloxy)-6,6-dimethoxyhexan-2-one* (**15**). Mercuric oxide (HgO; yellow) (1.06 g, 4.90 mmol) was added to a solution of ketone **14** (790 mg, 1.22 mmol) in boiling MeOH (33 mL). The hot solution was stirred vigorously while a solution of HgCl_2_ (1.00 g, 3.67 mmol) in dry MeOH (4 mL) was added over a period of 1 min, and stirring was continued while the suspension was refluxed for 15 min. The hot solution was filtered, the solid matter washed with MeOH, and the combined solutions concentrated *in vacuo* to give a syrup, which was dissolved in CH_2_Cl_2_ (25 mL). A white solid was filtered off and the filtrate washed successively with water, 10% aqueous KI (2 × 10 mL) and water (3 × 10 mL). The solution was dried over MgSO_4_ and the solvent was removed *in vacuo* to give a colorless oil. Column chromatography (petroleum ether-ethyl acetate = 5:1) provided ketone **15 **(577 mg, 81%) as a colourless oil. ^1^H-NMR (CDCl_3_) δ 7.32–7.07 (m, 20H), 4.64 (d, *J* = 10.8 Hz, 1H), 4.56 (d, *J* = 11.8 Hz, 1H), 4.54 (d, *J* = 11.3 Hz, 1H), 4.49–4.42 (m, 2H), 4.35 (d, *J* = 5.9 Hz, 1H), 4.28 (d, *J* = 11.9 Hz, 1H), 4.22 (d, *J* = 11.9 Hz, 1H), 4.12 (s, 2H), 4.07 (d, *J* = 4.9 Hz, 1H), 3.93 (t, *J* = 4.5 Hz, 1H), 3.65 (dd, *J* = 5.9, 4.3 Hz, 1H), 3.31 (s, 3H), 3.22 (s, 3H). ^13^C-NMR (CDCl_3_) δ 206.3, 138.5, 137.8, 137.7, 137.4, 128.5, 128.5, 128.43, 128.41, 128.38, 128.2, 128.0, 127.9, 127.8, 127.5, 105.5, 80.9, 80.0, 77.7, 74.8, 74.3, 74.3, 73.3, 73.2, 56.0, 54.7. [lit. [89] ^1^H-NMR (300 MHz; CDCl_3_) δ 7.50–7.15 (m, 20H), 4.72 (d, *J* = 11.0 Hz, 1H), 4.64 (d, *J* = 12.0 Hz, 1H), 4.62 (d, *J* = 11.0 Hz, 1H), 4.53 (m, 2H), 4.46 (d, *J* = 12.0 Hz, 1H), 4.43 (d, *J* = 6.3 Hz, 1H), 4.37 (d, *J* = 12.0 Hz, 1H), 4.31 (d, *J* = 12.0 Hz, 1H), 4.21 (s, 2H), 4.15 (d, *J* = 4.7 Hz, 1H), 4.00 (dd, *J* = 4.5, 4.7 Hz, 1H), 3.73 (dd, *J* = 4.5, 6.3 Hz, 1H), 3.40 (s, 3H), 3.31 (s, 3H); ^13^C-NMR (75 MHz; CDCl_3_) δ 206.3, 138.4, 137.7, 137.6, 137.3, 128.5, 128.45, 128.43, 128.39, 128.36, 128.2, 128.0, 127.8, 127.7, 127.5, 105.4, 80.8, 79.9, 77.6, 74.7, 74.3, 74.2, 73.3, 73.2, 56.0, 54.7].

*N-((3R,4S,5R)-1,3,4,5-Tetrakis(benzyloxy)-6,6-dimethoxyhexan-2-yl)hydroxylamine* (**16**). A stirred solution of ketone **15 **(592 mg, 1.01 mmol), hydroxylamine hydrochloride (211 mg, 3.03 mmol), pyridine (327 mL, 4.05 mmol) and EtOH (2.5 mL) was heated at 60 °C for 20 min. The solvent was removed *in vacuo* and the residue taken up in Et_2_O and washed with water. The organic layer was dried over MgSO_4_, filtered and the solvent was removed *in vacuo*. Column chromatography (petroleum ether-ethyl acetate = 3:1 containing 1% Et_3_N) provided oxime (546 mg, 90%) as a colourless oil and as a 1:1 mixture of *Z* and *E* isomers. The oxime (200 mg, 0.33 mmol) was redissolved in methanol (10 mL) and sodium cyanoborohydride (42 mg, 0.66 mmol) was added. 4 drops of methyl orange was added as the indicator. The resulting solution was carefully treated at room temperature with concentrated HCl/methanol (v/v = 1/10) to maintain the pH value between 3 and 4. After the completion of the reaction, the resulting solution was concentrated in *vacuo*. The residue was dissolved in CH_2_Cl_2_ (10 mL) and washed with 1 N NaOH (1 × 10 mL) and then brine (2 × 10 mL). After dried (MgSO_4_) and condensed under reduced pressure, the crude product was purified by column chromatography on silica gel (petroleum ether-ethyl acetate = 3:1 to 1:1) to afford isomers of hydroxylamine **16** (181 mg, 73% for 2 steps) as a colorless oil. ^1^H-NMR (CDCl_3_) δ 7.49–7.12 (m, 20H), 5.14 (br, 1H), 4.87 (d, *J* = 10.7 Hz, 2H), 4.83–4.71 (m, 3H), 4.64 (d, *J* = 10.5 Hz, 3H), 4.59 (s, 2H), 4.50 (d, *J* = 11.0 Hz, 2H), 4.38 (s, 3H), 4.08 (s, 1H), 3.99 (s, 1H), 3.70 (s, 2H), 3.64–3.52 (m, 2H), 3.46 (s, 3H), 3.36 (s, 2H), 3.25 (s, 3H), 3.20 (d, *J* = 7.0 Hz, 2H). ^13^C-NMR (CDCl_3_) δ 138.8, 138.5, 138.1, 128.4, 128.3, 128.1, 127.8, 127.6, 127.4, 105.4, 79.5, 78.3, 78.2, 75.0, 74.8, 73.9, 73.3, 68.4, 62.0, 56.0, 54.1. ESI-MS:* m/z* 602.96 [M+H]^+^

*(2R,3R,4S,5S)-3,4,5-tris(benzyloxy)-2-((benzyloxy)methyl)-2,3,4,5-Tetrahydropyridine 1-oxide (d-Glucopyranose-Derived Piperidine Nitrone* (**7**) *and (2S,3R,4S,5S)-3,4,5-tris(benzyloxy)-2-((benzyloxy)methyl)-2,3,4,5-Tetrahydropyridine 1-oxide (l-Idopyranose-Derived Piperidine Nitrone* (**17**). To a solution of hydroxylamine **16** (180 mg, 0.3 mmol) in CH_3_CN (6 mL) was added concentrated HCl (2 mL) and the resulting solution was stirred at room temperature for 2 h. The solution was diluted with EtOAc (10 mL) and then washed with H_2_O (2 × 20 mL) and saturated NaHCO_3_ solution (1 × 10 mL). The organic phase was dried (MgSO_4_) and concentrated under reduced pressure. The residue was purified by column chromatography on silica gel (petroleum ether: ethyl acetate = 1:1 to ethyl acetate) to afford d-glucopyranose-type nitrone** 7** (87 mg, 54%) and l-idopyranose-type nitrone **17** (44 mg, 27%) as yellow oils. 

*d-Glucopyranose-derived* *piperidine* *nitrone* (**7**). [α]_D_^14^ = +33.33° (*c* 1.2, CH_2_Cl_2_). IR (KBr, cm^−1^): 3031, 2870, 1454. ^1^H-NMR (CDCl_3_) δ 7.41–7.22 (m, 17H), 7.17 (d, *J* = 3.5 Hz, 2H), 7.16 (s, 1H), 7.06 (s, 1H), 4.86 (d, *J* = 4.1 Hz, 2H), 4.81 (s, 1H), 4.68 (s, 2H), 4.56 (d, *J* = 12.0 Hz, 1H), 4.47 (d, *J* = 11.0 Hz, 1H), 4.38 (s, 1H), 4.35 (d, *J* = 4.1 Hz, 1H), 4.26 (dd, *J* = 5.5, 2.2 Hz, 1H), 4.17–4.08 (m, 1H), 3.85 (dd, *J* = 9.5, 7.9 Hz, 1H), 3.76 (d, *J* = 8.3 Hz, 1H), 3.63 (d, *J* = 10.0 Hz, 1H). ^13^C-NMR (CDCl_3_) δ 137.9, 137.8, 137.6 137.0, 135.3, 128.7, 128.5, 128.4, 128.3, 128.1, 128.0, 128.0, 127.8, 81.0, 76.1, 75.1, 73.8, 73.3, 73.2, 72.4, 64.6. HRMS-ESI (*m/z*): calcd for C_34_H_36_NO_5_ [M+H]^+^ 538.25880, found 538.25879.

*l-Idopyranose-derived Piperidine Nitrone* (**17**). [α]_D_^14^ = −0.1° (*c* 1.0, CH_2_Cl_2_). IR (KBr, cm^−1^): 3031, 2825, 1454. ^1^H-NMR (CDCl_3_) δ 7.40–7.33 (m, 12H), 7.32–7.28 (m, 6H), 7.26 (d, *J* = 6.9 Hz, 2H), 7.10 (d, *J* = 2.7 Hz, 1H), 4.89 (d, *J* = 11.3 Hz, 1H), 4.80 (t, *J* = 12.4 Hz, 2H), 4.67 (d, *J* = 11.6 Hz, 2H), 4.60 (t, *J* = 10.4 Hz, 2H), 4.53 (d, *J* = 12.0 Hz, 1H), 4.51–4.48 (m, 1H), 4.30 (s, 1H), 4.17 (dd, *J* = 9.9, 3.4 Hz, 1H), 4.14 (d, *J* = 8.6 Hz, 1H), 4.02 (s, 1H), 4.01–3.97 (m, 1H). ^13^C-NMR (CDCl_3_) δ 138.1, 137.8, 137.43, 135.6, 128.6, 128.5, 128.5, 128.3, 128.1, 128.0, 127.9, 127.8, 127.6, 77.0, 76.4, 75.1, 74.4, 73.7, 73.6, 71.7, 70.0, 65.2. HRMS-ESI (*m/z*): calcd for C_34_H_36_NO_5_ [M+H]^+^ 538.25880, found 538.25879.

## 4. Conclusions

In summary, d-glucopyranose- and L-idopyranose-derived piperidine nitrones have been synthesized in good overall yields through six-step reaction sequence starting from the readily available 2,3,4,6-tetra-*O*-benzyl-d-glucopyranose . The method is efficient and could be general for the synthesis of aldohexose-derived piperidine nitrones which are precursors of piperidine iminosugars. Based on this procedure, many other hexopyranose-derived cyclic nitrones can be prepared, therefore this method should prove significant for future work on the design, synthesis and discovery of glycosidase inhibitors with better selectivity and potency.

## Supplementary Materials

Supplementary materials can be accessed at: http://www.mdpi.com/1420-3049/18/5/6021/s1.
