# Supplementary material for: An Efficient Synthesis of Aldohexose-Derived Piperidine Nitrones: Precursors of Piperidine Iminosugars
Source: Molecules. 2013 May 21;18(5):6021–34. doi: 10.3390/molecules18056021 (PMC6270483; doi:10.3390/molecules18056021)
Supplement: Supplementary file 1 [file molecules-18-06021-s001.pdf]

Supplementary Materials

Section A: NOESY of nitrone 7 and nitrone 17

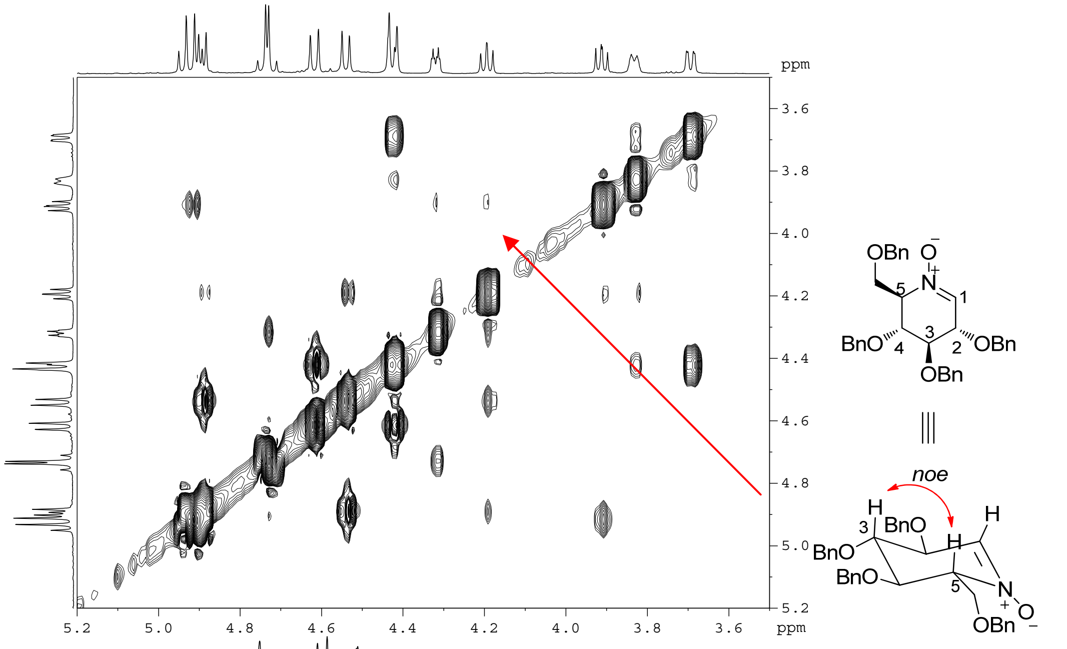

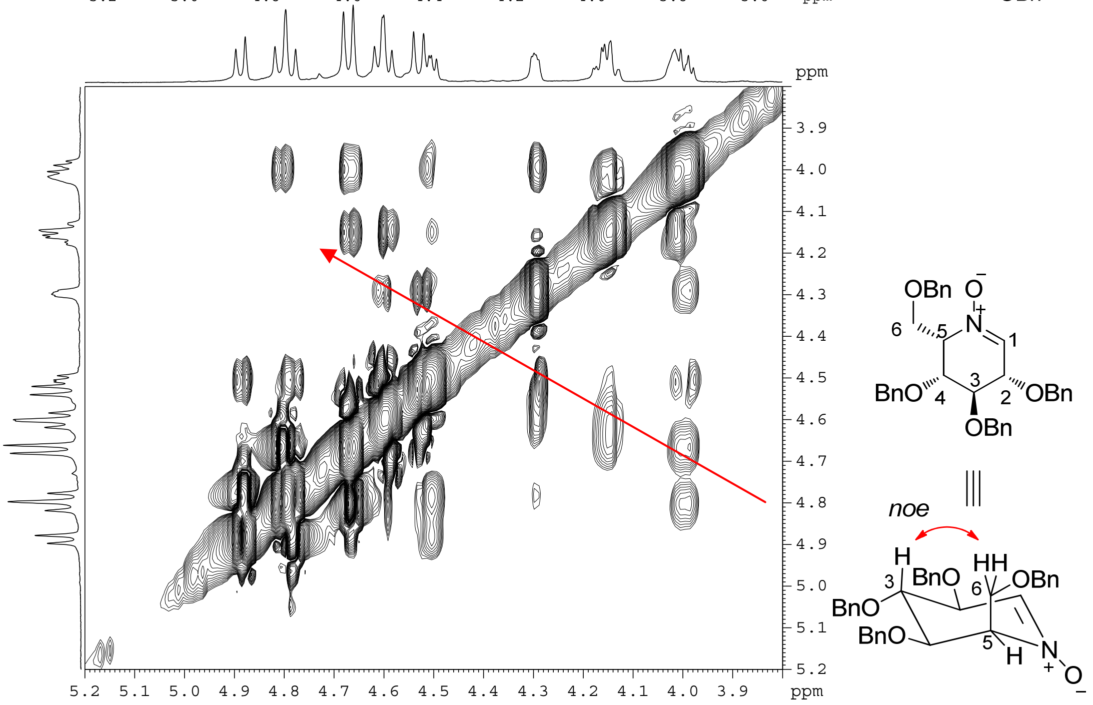

Section B: Spectra of Compounds

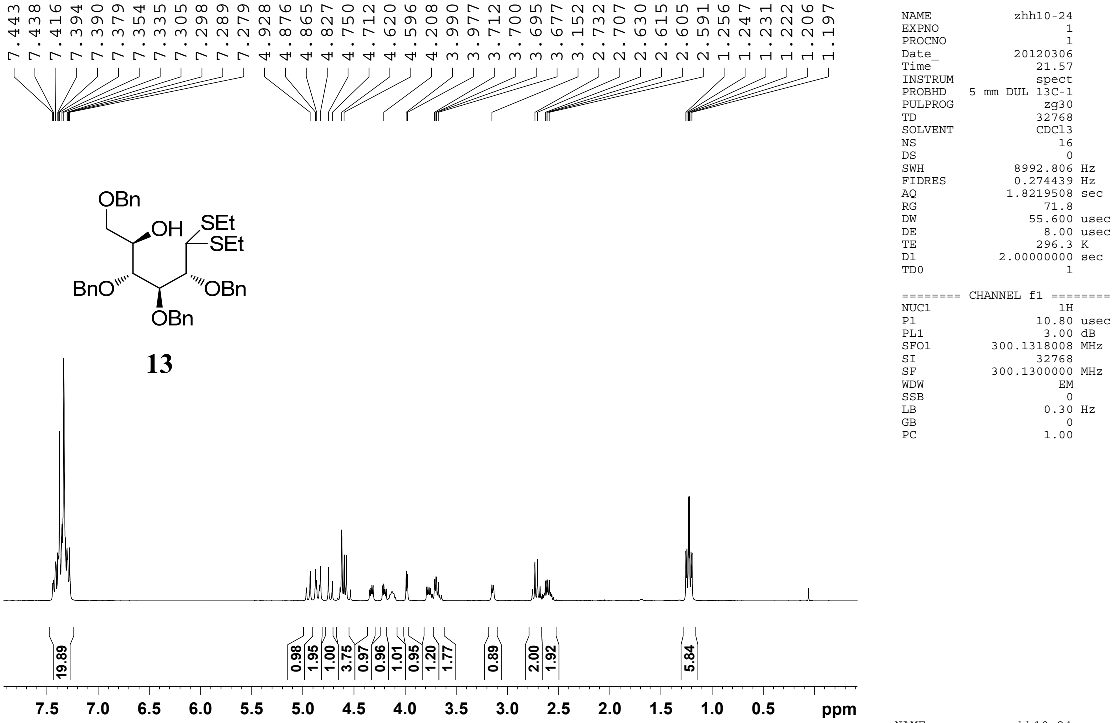

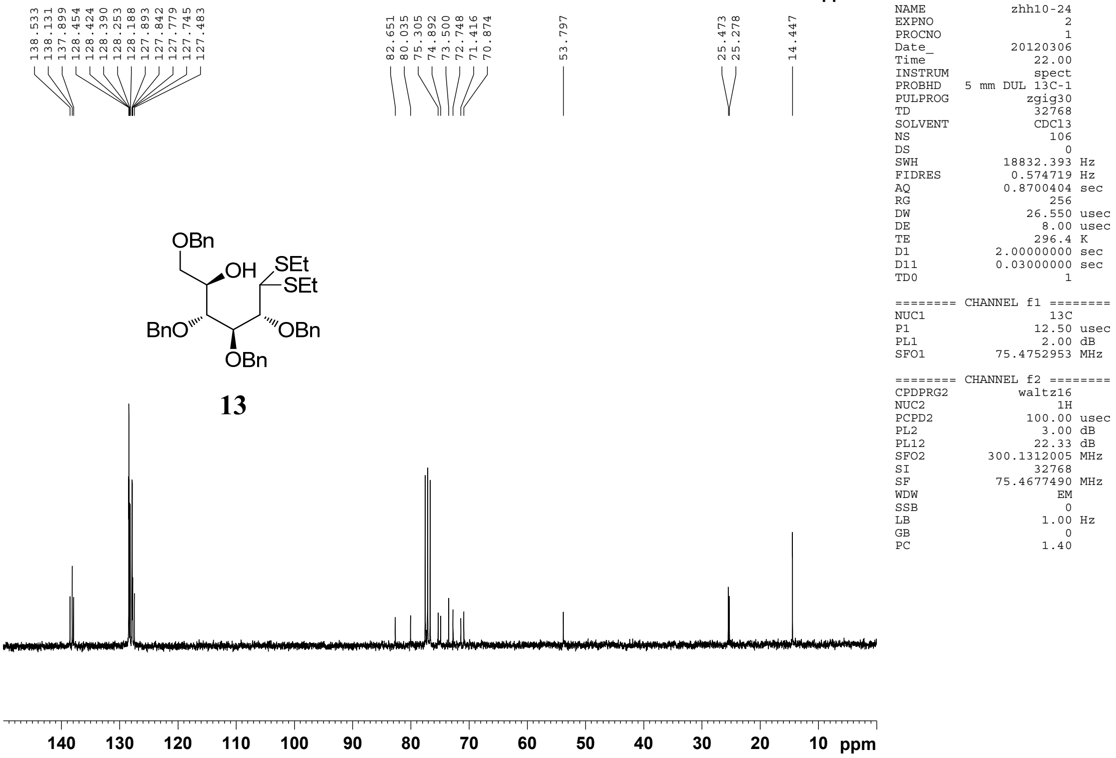

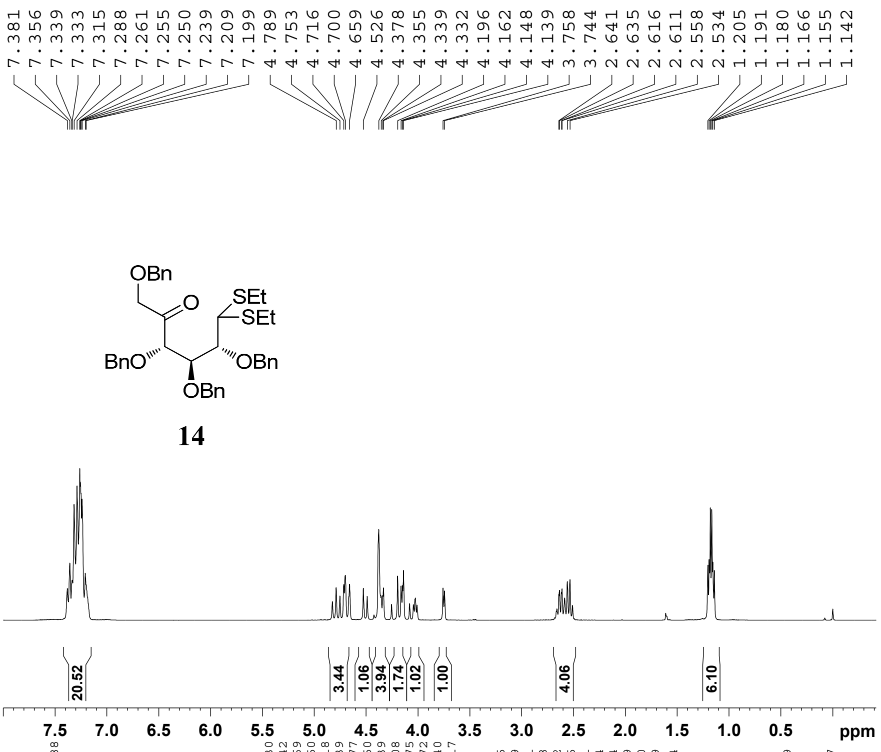


```
NAME            zhh10-25
EXPNO                  1
PROCNO                 1
Date_           20120309
Time               12.29
INSTRUM            spect
PROBHD   5 mm DUL 13C-1
PULPROG             zg30
TD                 65536
SOLVENT            CDCl3
NS                    16
DS                     0
SWH             8992.806 Hz
FIDRES          0.137219 Hz
AQ             3.6438515 sec
RG                   128
DW                55.600 usec
DE                  8.00 usec
TE                 295.4 K
D1            1.00000000 sec
TD0                    1

======== CHANNEL f1 ========
NUC1                  1H
P1                 10.80 usec
PL1                 3.00 dB
SFO1         300.1324010 MHz
SI                 32768
SF           300.1300134 MHz
WDW                   EM
SSB                    0
LB                  0.30 Hz
GB                     0
PC                  1.00
```

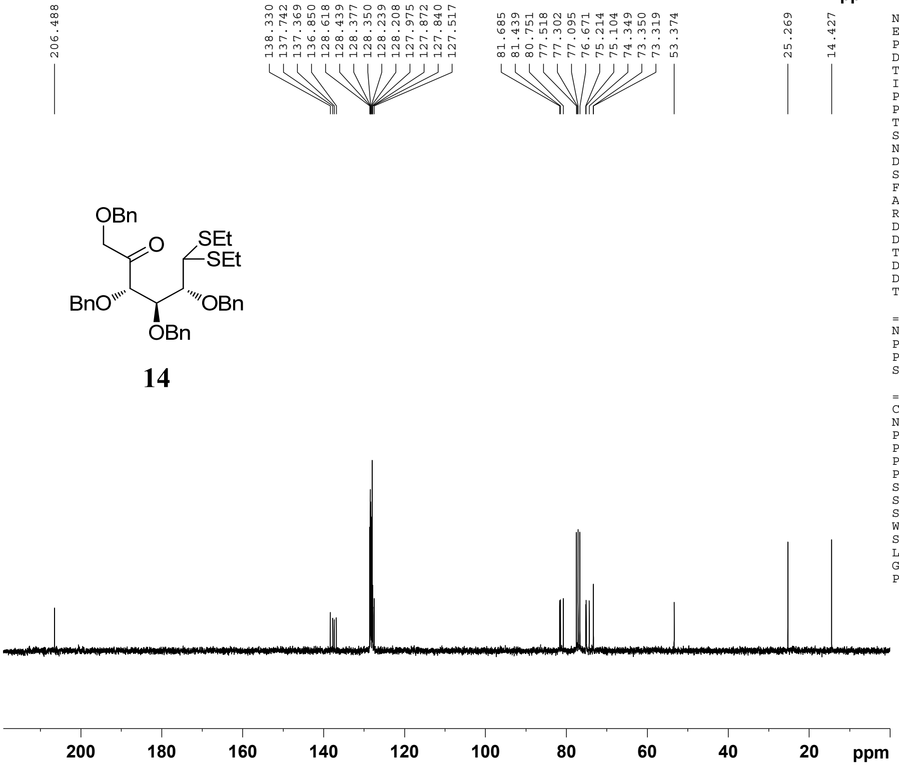


```
NAME            zhh10-25
EXPNO                  2
PROCNO                 1
Date_           20120309
Time               12.32
INSTRUM            spect
PROBHD   5 mm DUL 13C-1
PULPROG           zgpg30
TD                 65536
SOLVENT            CDCl3
NS                    61
DS                     4
SWH            17985.611 Hz
FIDRES          0.274439 Hz
AQ             1.8219508 sec
RG                 574.7
DW                27.800 usec
DE                  6.50 usec
TE                 295.7 K
D1            2.00000000 sec
D11           0.03000000 sec
TD0                    1

======== CHANNEL f1 ========
NUC1                 13C
P1                 12.50 usec
PL1                 2.00 dB
SFO1          75.4752953 MHz

======== CHANNEL f2 ========
CPDPRG2          waltz16
NUC2                  1H
PCPD2             100.00 usec
PL2                 3.00 dB
PL12               22.33 dB
PL13               23.00 dB
SFO2         300.1312005 MHz
SI                 32768
SF            75.4677490 MHz
WDW                   EM
SSB                    0
LB                  1.00 Hz
GB                     0
PC                  1.40
```

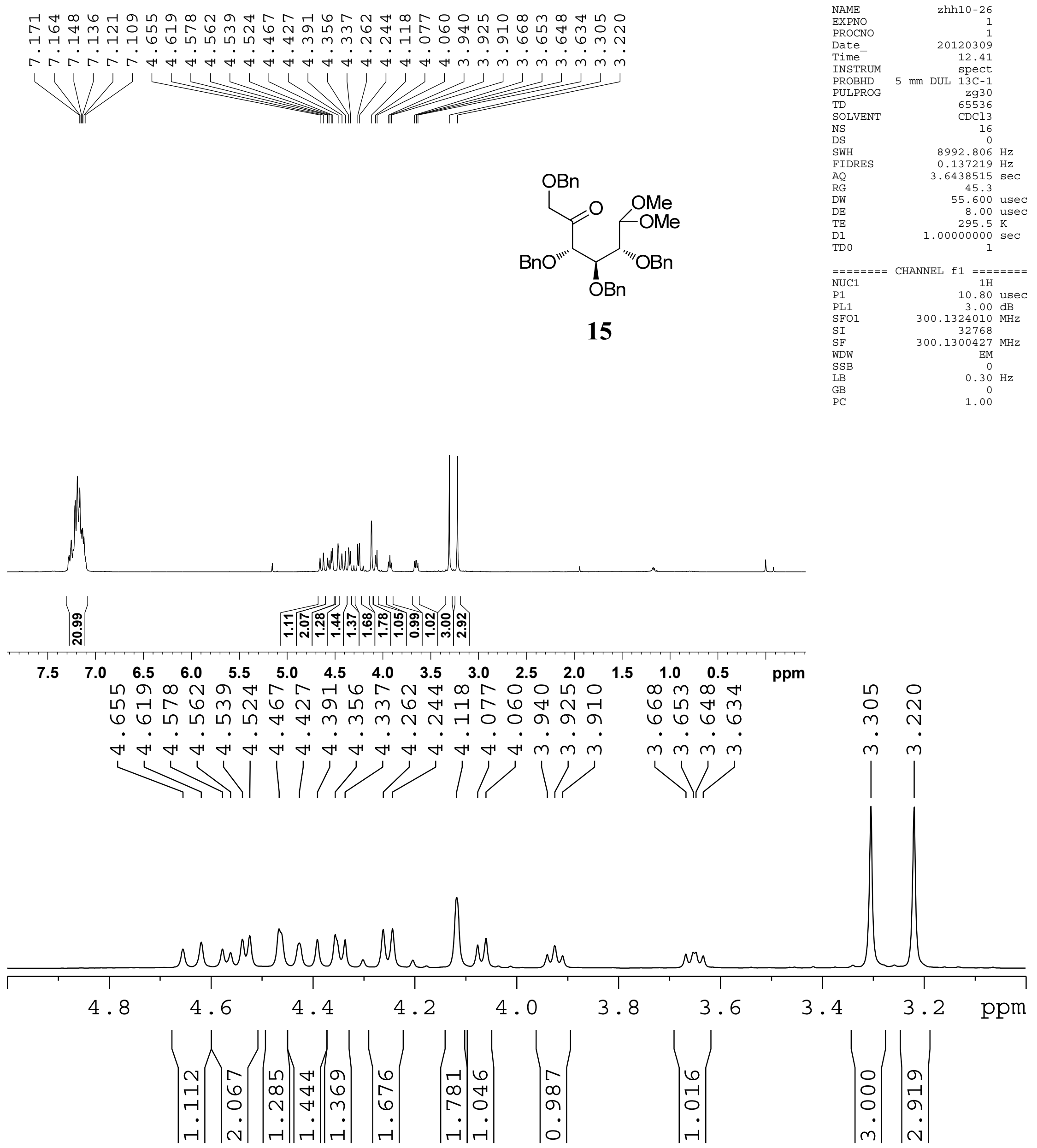
NAME zhh10-26
EXPNO 1
PROCNO 1
Date_ 20120309
Time 12.41
INSTRUM spect
PROBHD 5 mm DUL 13C-1
PULPROG zg30
TD 65536
SOLVENT CDCl3
NS 16
DS 0
SWH 8992.806 Hz
FIDRES 0.137219 Hz
AQ 3.6438515 sec
RG 45.3
DW 55.600 usec
DE 8.00 usec
TE 295.5 K
D1 1.00000000 sec
TD0 1
======== CHANNEL f1 ========
NUC1 1H
P1 10.80 usec
PL1 3.00 dB
SFO1 300.1324010 MHz
SI 32768
SF 300.1300427 MHz
WDW EM
SSB 0
LB 0.30 Hz
GB 0
PC 1.00
OBn
O
OMe
OMe
BnO
OBn
OBn
15
ppm

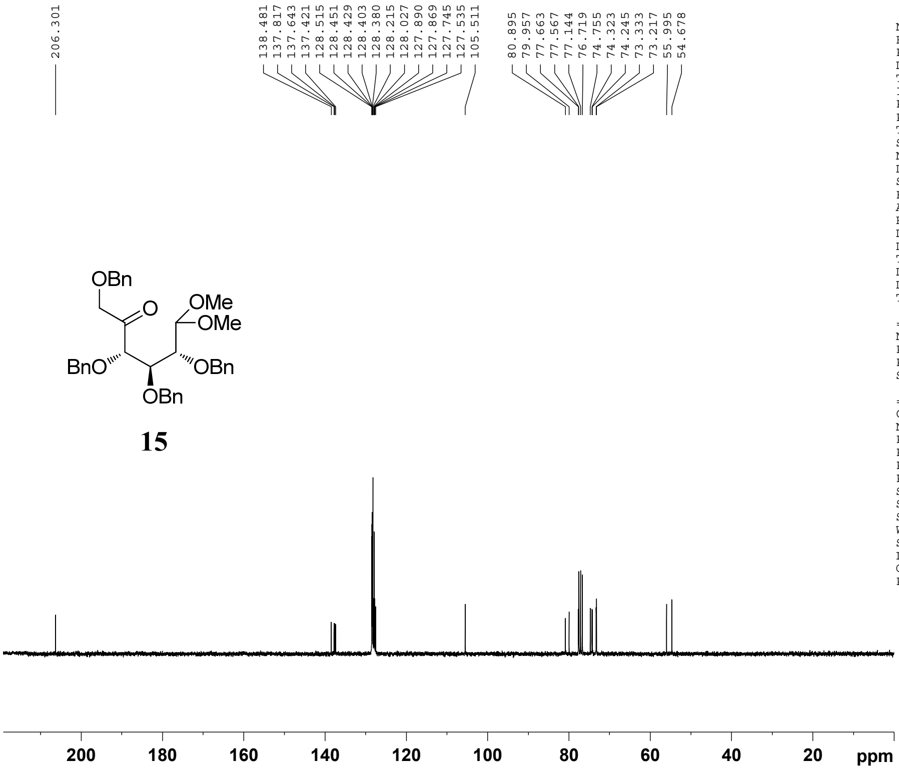


```
NAME           zhh10-26
EXPNO                 2
PROCNO                1
Date_          20120309
Time              12.43
INSTRUM           spect
PROBHD   5 mm DUL 13C-1
PULPROG          zgpg30
TD                65536
SOLVENT           CDCl3
NS                   53
DS                    4
SWH           17985.611 Hz
FIDRES         0.274439 Hz
AQ            1.8219508 sec
RG                812.7
DW               27.800 usec
DE                 6.50 usec
TE                295.8 K
D1           2.00000000 sec
D11          0.03000000 sec
TD0                   1

======== CHANNEL f1 ========
NUC1                13C
P1                12.50 usec
PL1                2.00 dB
SFO1         75.4752953 MHz

======== CHANNEL f2 ========
CPDPRG2         waltz16
NUC2                 1H
PCPD2            100.00 usec
PL2                3.00 dB
PL12              22.33 dB
PL13              23.00 dB
SFO2        300.1312005 MHz
SI                32768
SF           75.4677490 MHz
WDW                  EM
SSB                   0
LB                 1.00 Hz
GB                    0
PC                 1.40
```

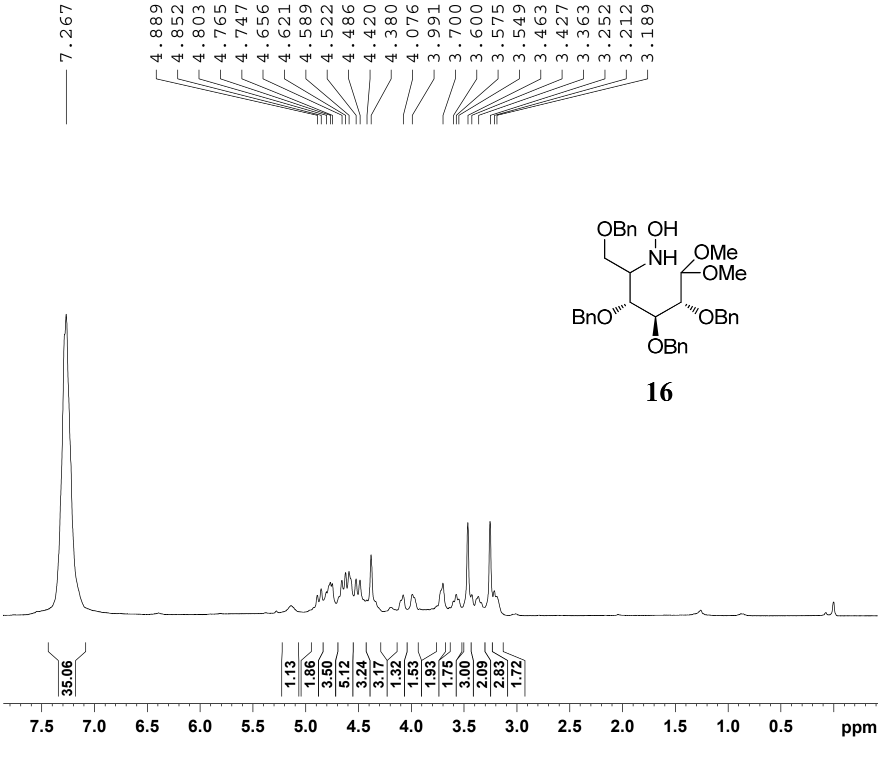


```
NAME           zhh10-28
EXPNO                10
PROCNO                1
Date_          20120312
Time               9.02
INSTRUM           spect
PROBHD   5 mm DUL 13C-1
PULPROG            zg30
TD                65536
SOLVENT           CDCl3
NS                   16
DS                    0
SWH            8992.806 Hz
FIDRES         0.137219 Hz
AQ            3.6438515 sec
RG                  128
DW               55.600 usec
DE                 8.00 usec
TE                295.7 K
D1           1.00000000 sec
TD0                   1

======== CHANNEL f1 ========
NUC1                 1H
P1                10.80 usec
PL1                3.00 dB
SFO1        300.1324010 MHz
SI                32768
SF          300.1300108 MHz
WDW                  EM
SSB                   0
LB                 0.30 Hz
GB                    0
PC                 1.00
```

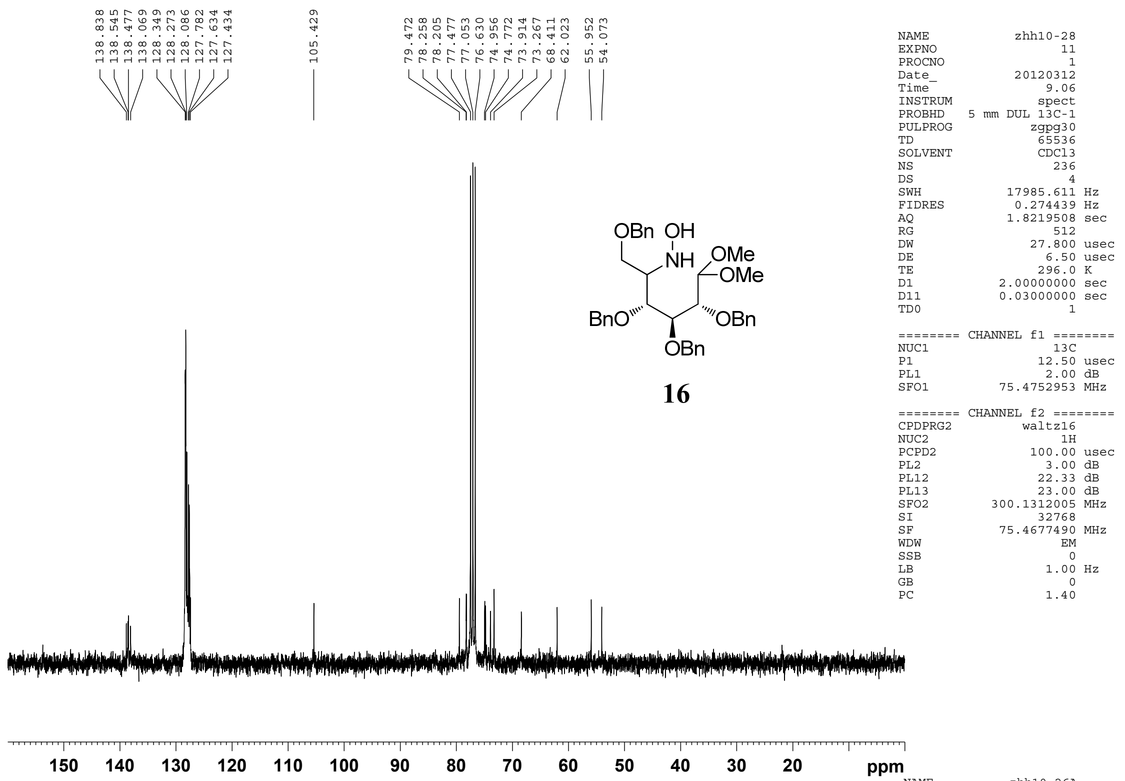
138.838
138.545
138.477
138.069
128.349
128.273
128.086
127.782
127.634
127.434
105.429
79.472
78.258
78.205
77.477
77.053
76.630
74.956
74.772
73.914
73.267
68.411
62.023
55.952
54.073
OBn OH
NH
OMe
OMe
BnO
OBn
OBn
16
150 140 130 120 110 100 90 80 70 60 50 40 30 20 ppm
NAME zhh10-28
EXPNO 11
PROCNO 1
Date_ 20120312
Time 9.06
INSTRUM spect
PROBHD 5 mm DUL 13C-1
PULPROG zgpg30
TD 65536
SOLVENT CDCl3
NS 236
DS 4
SWH 17985.611 Hz
FIDRES 0.274439 Hz
AQ 1.8219508 sec
RG 512
DW 27.800 usec
DE 6.50 usec
TE 296.0 K
D1 2.00000000 sec
D11 0.03000000 sec
TD0 1
======== CHANNEL f1 ========
NUC1 13C
P1 12.50 usec
PL1 2.00 dB
SFO1 75.4752953 MHz
======== CHANNEL f2 ========
CPDPRG2 waltz16
NUC2 1H
PCPD2 100.00 usec
PL2 3.00 dB
PL12 22.33 dB
PL13 23.00 dB
SFO2 300.1312005 MHz
SI 32768
SF 75.4677490 MHz
WDW EM
SSB 0
LB 1.00 Hz
GB 0
PC 1.40

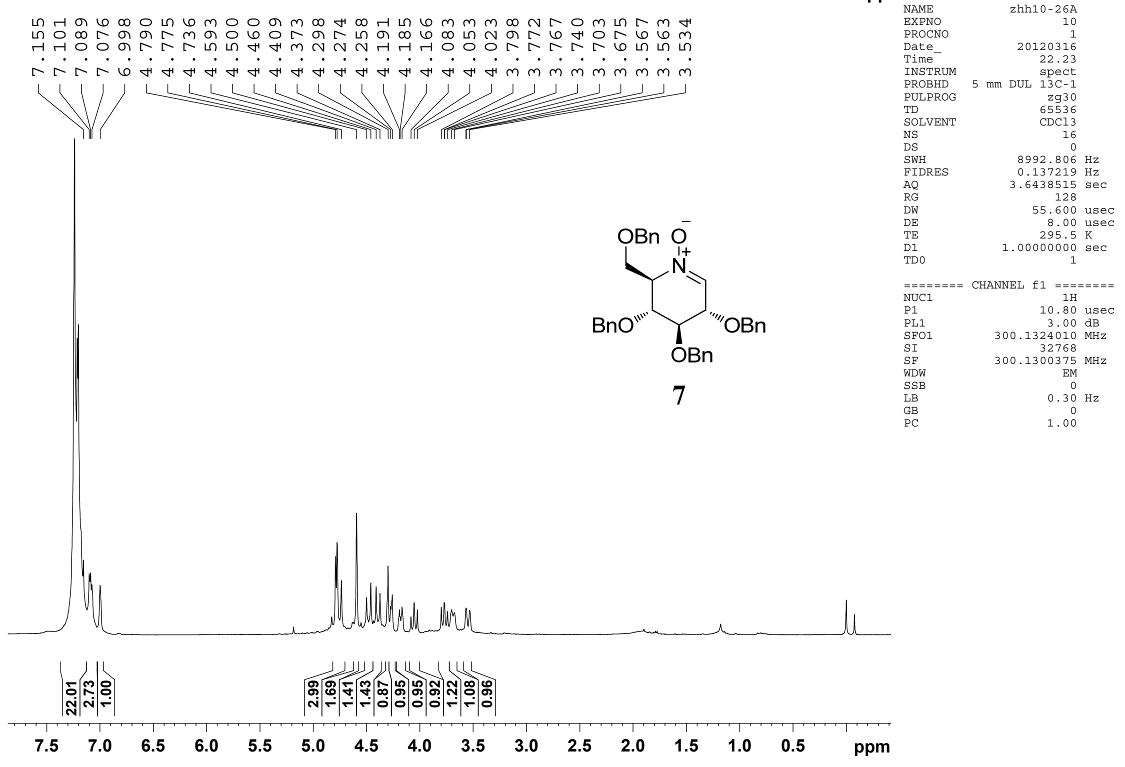
7.155
7.101
7.089
7.076
6.998
4.790
4.775
4.736
4.593
4.500
4.460
4.409
4.373
4.298
4.274
4.258
4.191
4.185
4.166
4.083
4.053
4.023
3.798
3.772
3.767
3.740
3.703
3.675
3.567
3.563
3.534
OBn O
N+
BnO
OBn
OBn
7
22.01
2.73
1.00
2.99
1.69
1.41
1.43
0.87
0.95
0.95
0.92
1.22
1.08
0.96
7.5 7.0 6.5 6.0 5.5 5.0 4.5 4.0 3.5 3.0 2.5 2.0 1.5 1.0 0.5 ppm
NAME zhh10-26A
EXPNO 10
PROCNO 1
Date_ 20120316
Time 22.23
INSTRUM spect
PROBHD 5 mm DUL 13C-1
PULPROG zg30
TD 65536
SOLVENT CDCl3
NS 16
DS 0
SWH 8992.806 Hz
FIDRES 0.137219 Hz
AQ 3.6438515 sec
RG 128
DW 55.600 usec
DE 8.00 usec
TE 295.5 K
D1 1.00000000 sec
TD0 1
======== CHANNEL f1 ========
NUC1 1H
P1 10.80 usec
PL1 3.00 dB
SFO1 300.1324010 MHz
SI 32768
SF 300.1300375 MHz
WDW EM
SSB 0
LB 0.30 Hz
GB 0
PC 1.00

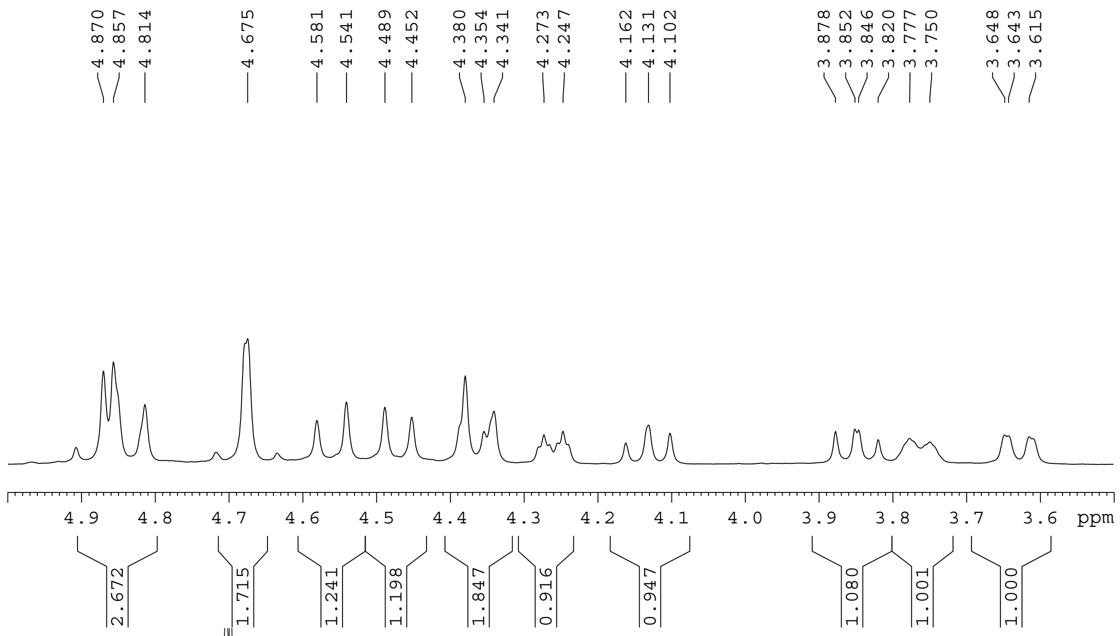

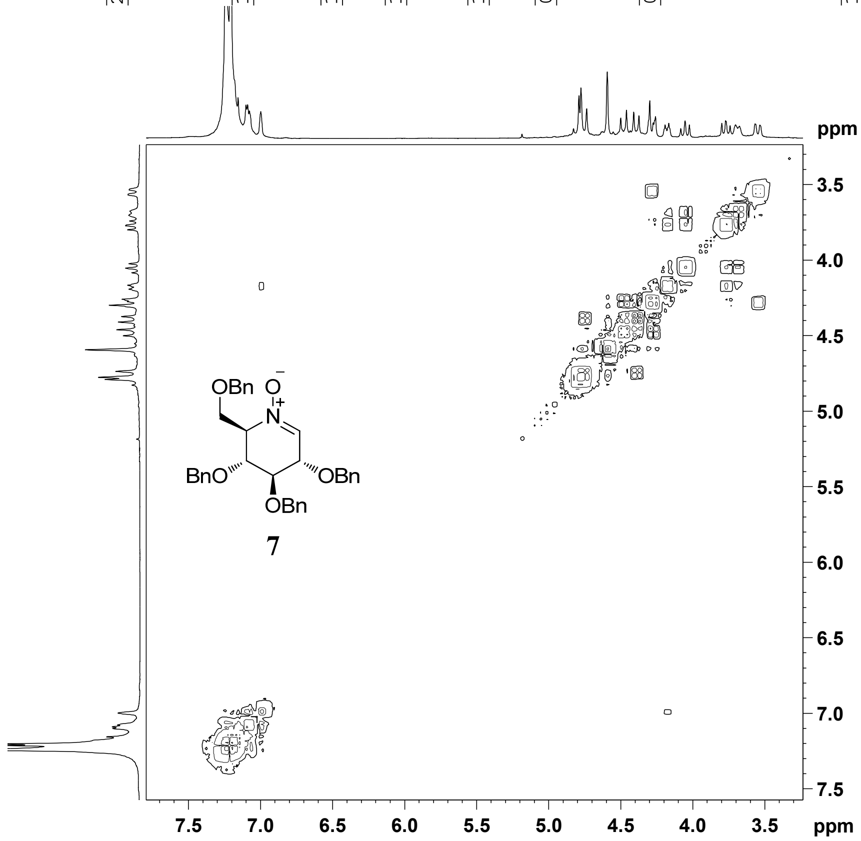


```
NAME          zhh10-26A
EXPNO                11
PROCNO                1
Date_          20120316
Time              22.23
INSTRUM           spect
PROBHD   5 mm DUL 13C-1
PULPROG       cosygpqf
TD                 2048
SOLVENT           CDCl3
NS                    4
DS                    8
SWH            3086.420 Hz
FIDRES         1.507041 Hz
AQ            0.3318260 sec
RG                 45.3
DW              162.000 usec
DE                 8.00 usec
TE                295.5 K
D0           0.00000300 sec
D1           1.46477306 sec
D13          0.00000400 sec
D16          0.00020000 sec
IN0          0.00032400 sec

======== CHANNEL f1 ========
NUC1                 1H
P0                10.80 usec
P1                10.80 usec
PL1                3.00 dB
SFO1        300.1313753 MHz

====== GRADIENT CHANNEL =====
GPNAM1         SINE.100
GPZ1              10.00 %
P16             1000.00 usec
ND0                   1
TD                   57
SFO1           300.1314 MHz
FIDRES        54.147717 Hz
SW               10.284 ppm
FnMODE               QF
SI                 1024
SF          300.1300375 MHz
WDW                SINE
SSB                   0
LB                 0.00 Hz
GB                    0
PC                 1.40
SI                 1024
MC2                  QF
SF          300.1300375 MHz
WDW                SINE
SSB                   0
LB                 0.00 Hz
GB                    0
```

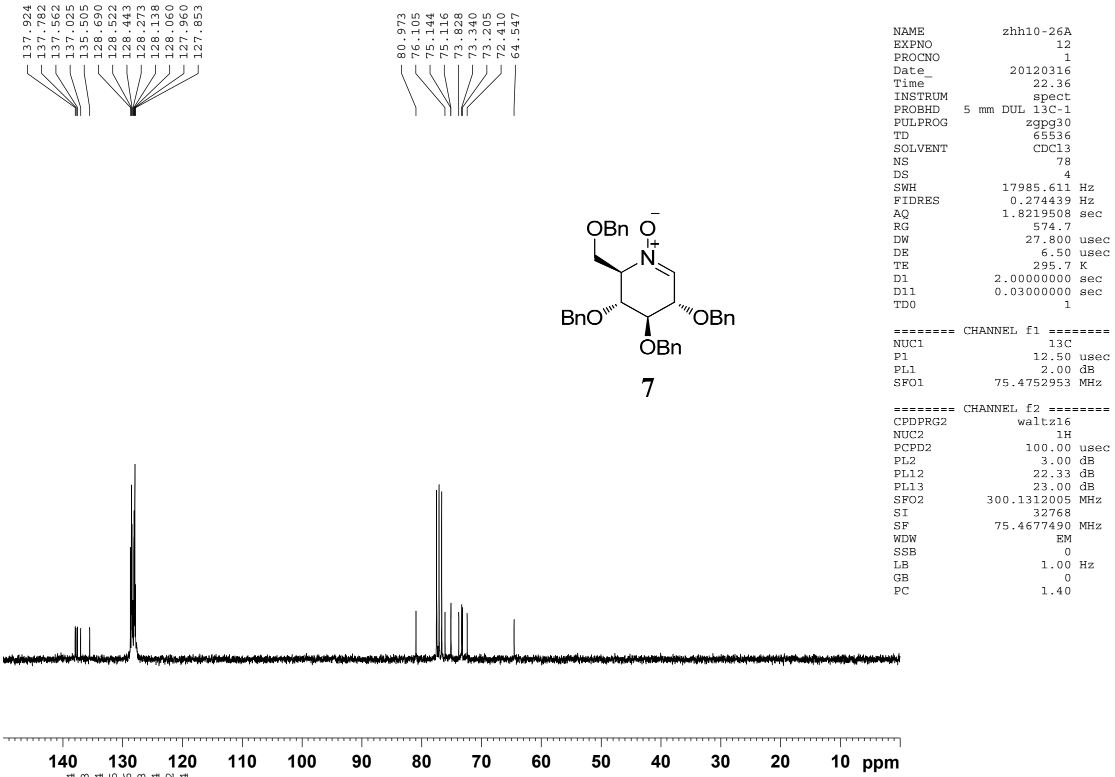
137.924
137.782
137.562
137.025
135.505
128.690
128.522
128.443
128.273
128.138
128.060
127.960
127.853
80.973
76.105
75.144
75.116
73.828
73.340
73.205
72.410
64.547
OBn
O
N
BnO
OBn
OBn
7
NAME zhh10-26A
EXPNO 12
PROCNO 1
Date_ 20120316
Time 22.36
INSTRUM spect
PROBHD 5 mm DUL 13C-1
PULPROG zgpg30
TD 65536
SOLVENT CDCl3
NS 78
DS 4
SWH 17985.611 Hz
FIDRES 0.274439 Hz
AQ 1.8219508 sec
RG 574.7
DW 27.800 usec
DE 6.50 usec
TE 295.7 K
D1 2.00000000 sec
D11 0.03000000 sec
TD0 1
======== CHANNEL f1 ========
NUC1 13C
P1 12.50 usec
PL1 2.00 dB
SFO1 75.4752953 MHz
======== CHANNEL f2 ========
CPDPRG2 waltz16
NUC2 1H
PCPD2 100.00 usec
PL2 3.00 dB
PL12 22.33 dB
PL13 23.00 dB
SFO2 300.1312005 MHz
SI 32768
SF 75.4677490 MHz
WDW EM
SSB 0
LB 1.00 Hz
GB 0
PC 1.40
140 130 120 110 100 90 80 70 60 50 40 30 20 10 ppm

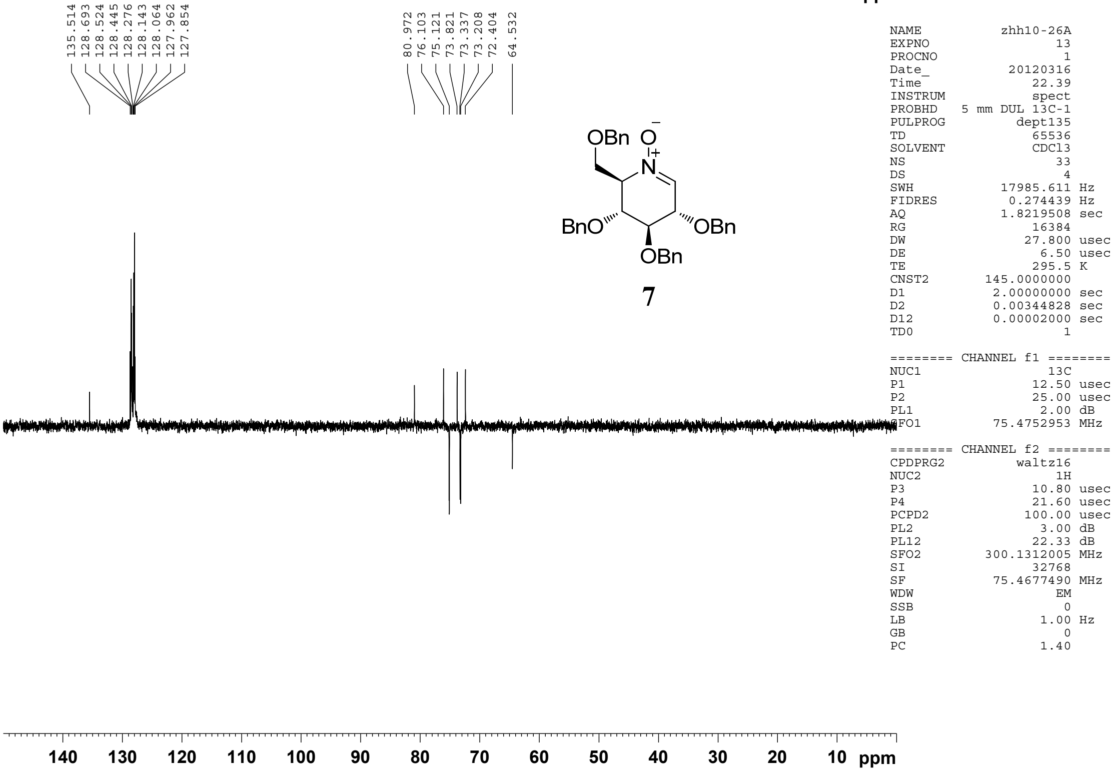
135.514
128.693
128.524
128.445
128.276
128.143
128.064
127.962
127.854
80.972
76.103
75.121
73.821
73.337
73.208
72.404
64.532
OBn
O
N
BnO
OBn
OBn
7
NAME zhh10-26A
EXPNO 13
PROCNO 1
Date_ 20120316
Time 22.39
INSTRUM spect
PROBHD 5 mm DUL 13C-1
PULPROG dept135
TD 65536
SOLVENT CDCl3
NS 33
DS 4
SWH 17985.611 Hz
FIDRES 0.274439 Hz
AQ 1.8219508 sec
RG 16384
DW 27.800 usec
DE 6.50 usec
TE 295.5 K
CNST2 145.0000000
D1 2.00000000 sec
D2 0.00344828 sec
D12 0.00002000 sec
TD0 1
======== CHANNEL f1 ========
NUC1 13C
P1 12.50 usec
P2 25.00 usec
PL1 2.00 dB
SFO1 75.4752953 MHz
======== CHANNEL f2 ========
CPDPRG2 waltz16
NUC2 1H
P3 10.80 usec
P4 21.60 usec
PCPD2 100.00 usec
PL2 3.00 dB
PL12 22.33 dB
SFO2 300.1312005 MHz
SI 32768
SF 75.4677490 MHz
WDW EM
SSB 0
LB 1.00 Hz
GB 0
PC 1.40
140 130 120 110 100 90 80 70 60 50 40 30 20 10 ppm

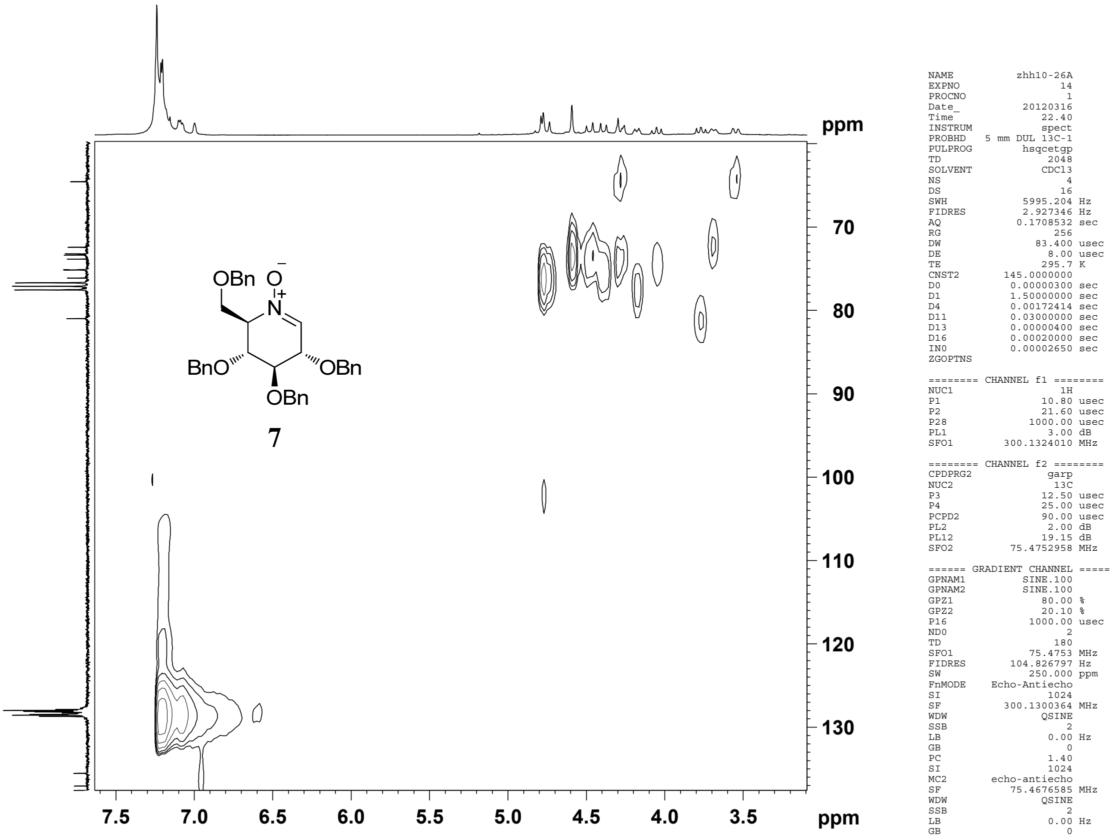
ppm
70
80
90
100
110
120
130
7.5 7.0 6.5 6.0 5.5 5.0 4.5 4.0 3.5 ppm
OBn O
N
BnO
OBn
OBn
7
NAME zhh10-26A
EXPNO 14
PROCNO 1
Date_ 20120316
Time 22.40
INSTRUM spect
PROBHD 5 mm DUL 13C-1
PULPROG hsqcetgp
TD 2048
SOLVENT CDCl3
NS 4
DS 16
SWH 5995.204 Hz
FIDRES 2.927346 Hz
AQ 0.1708532 sec
RG 256
DW 83.400 usec
DE 8.00 usec
TE 295.7 K
CNST2 145.0000000
D0 0.00000300 sec
D1 1.50000000 sec
D4 0.00172414 sec
D11 0.03000000 sec
D13 0.00000400 sec
D16 0.00020000 sec
IN0 0.00002650 sec
ZGOPTNS
======== CHANNEL f1 ========
NUC1 1H
P1 10.80 usec
P2 21.60 usec
P28 1000.00 usec
PL1 3.00 dB
SFO1 300.1324010 MHz
======== CHANNEL f2 ========
CPDPRG2 garp
NUC2 13C
P3 12.50 usec
P4 25.00 usec
PCPD2 90.00 usec
PL2 2.00 dB
PL12 19.15 dB
SFO2 75.4752958 MHz
====== GRADIENT CHANNEL =====
GPNAM1 SINE.100
GPNAM2 SINE.100
GPZ1 80.00 %
GPZ2 20.10 %
P16 1000.00 usec
ND0 2
TD 180
SFO1 75.4753 MHz
FIDRES 104.826797 Hz
SW 250.000 ppm
FnMODE Echo-Antiecho
SI 1024
SF 300.1300364 MHz
WDW QSINE
SSB 2
LB 0.00 Hz
GB 0
PC 1.40
SI 1024
MC2 echo-antiecho
SF 75.4676585 MHz
WDW QSINE
SSB 2
LB 0.00 Hz
GB 0

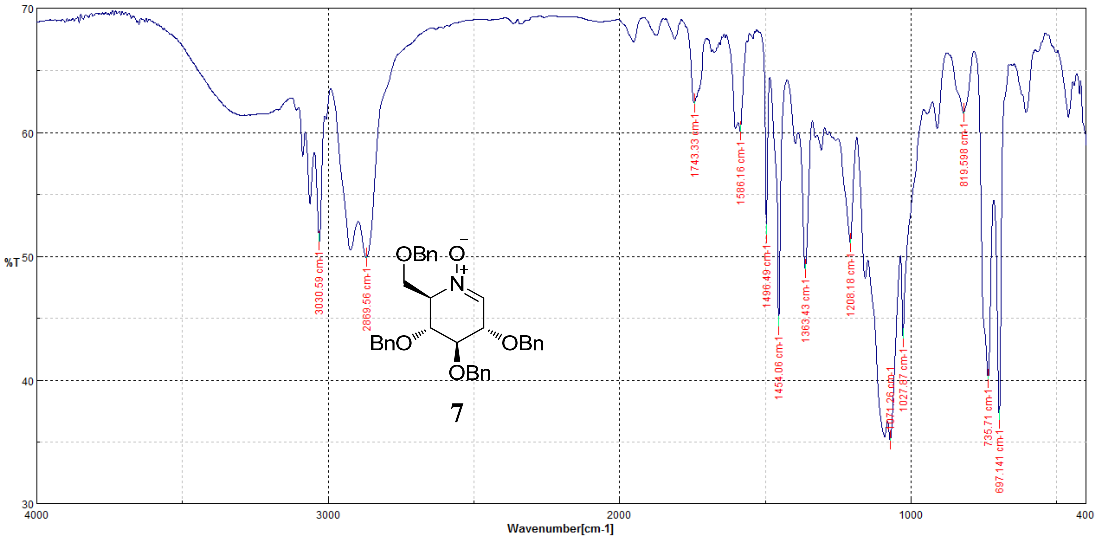
70
60
%T 50
40
30
4000 3000 2000 1000 400
Wavenumber[cm-1]
3030.59 cm-1
2869.56 cm-1
1743.33 cm-1
1586.16 cm-1
1496.49 cm-1
1454.06 cm-1
1363.43 cm-1
1208.18 cm-1
1071.26 cm-1
1027.87 cm-1
819.598 cm-1
735.71 cm-1
697.141 cm-1
OBn O
N
BnO
OBn
OBn
7

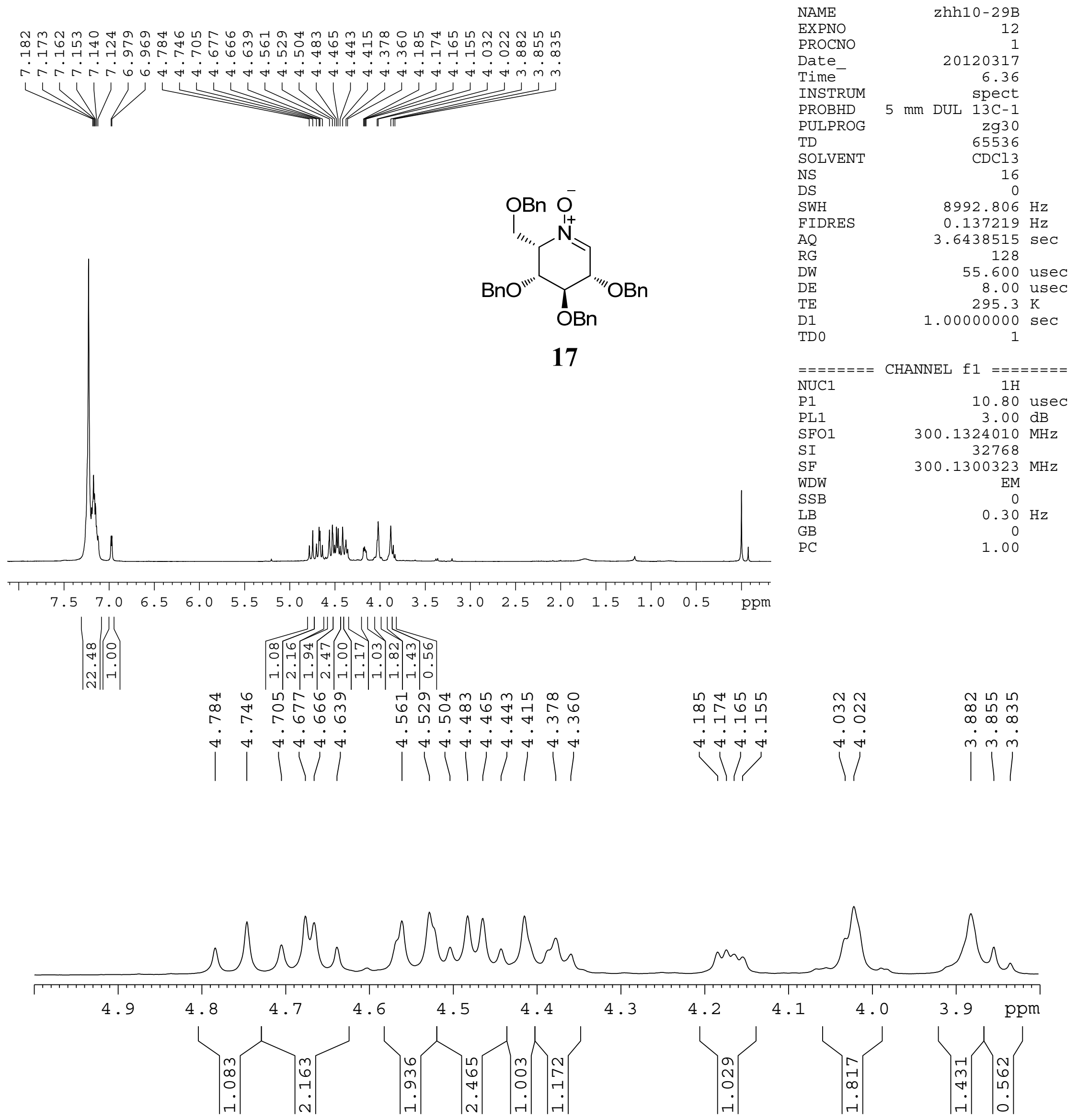
OBn
BnO
OBn
OBn
17
NAME zhh10-29B
EXPNO 12
PROCNO 1
Date_ 20120317
Time 6.36
INSTRUM spect
PROBHD 5 mm DUL 13C-1
PULPROG zg30
TD 65536
SOLVENT CDCl3
NS 16
DS 0
SWH 8992.806 Hz
FIDRES 0.137219 Hz
AQ 3.6438515 sec
RG 128
DW 55.600 usec
DE 8.00 usec
TE 295.3 K
D1 1.00000000 sec
TD0 1
======== CHANNEL f1 ========
NUC1 1H
P1 10.80 usec
PL1 3.00 dB
SFO1 300.1324010 MHz
SI 32768
SF 300.1300323 MHz
WDW EM
SSB 0
LB 0.30 Hz
GB 0
PC 1.00
ppm

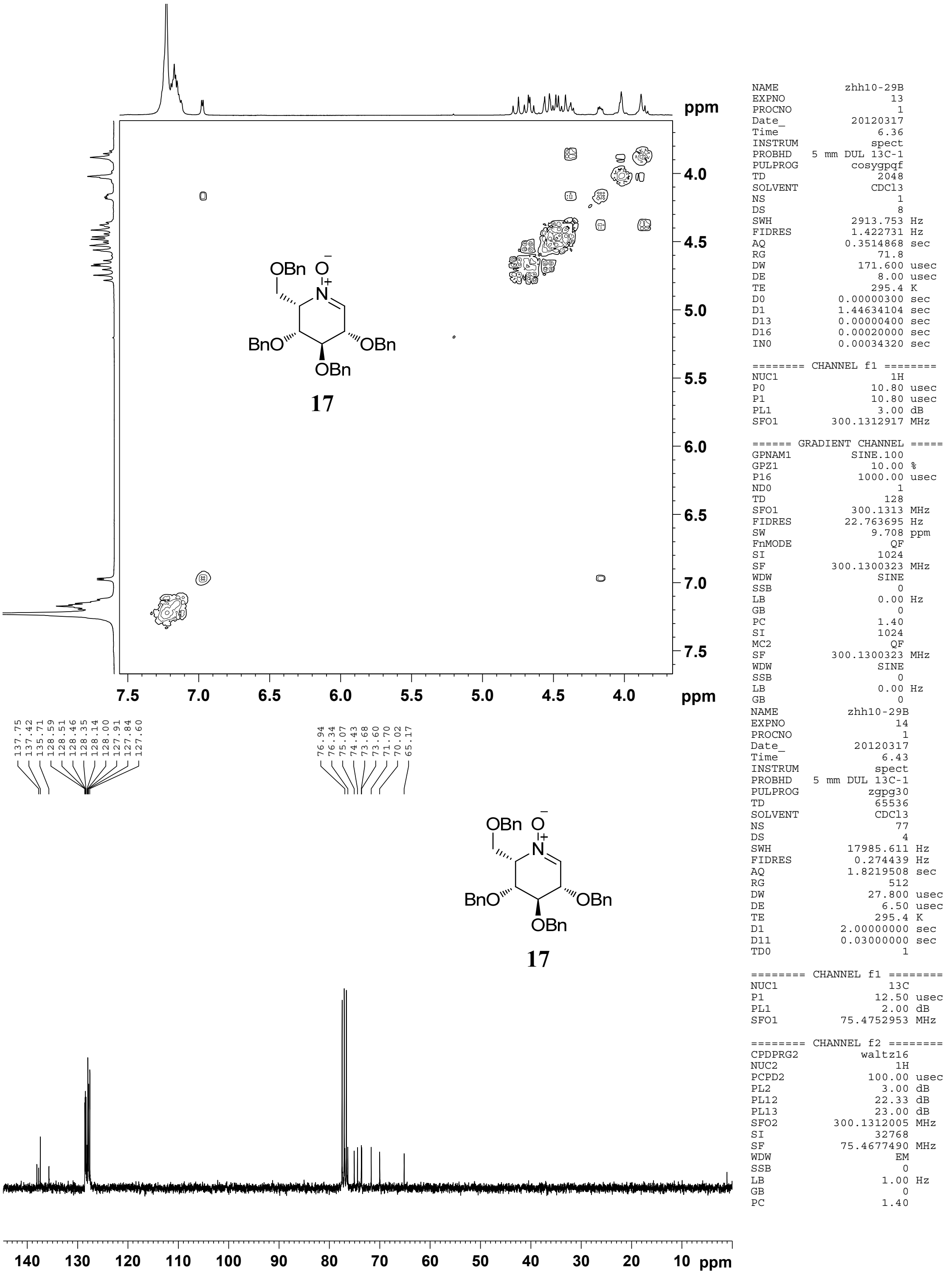
ppm
4.0
4.5
5.0
5.5
6.0
6.5
7.0
7.5
7.5 7.0 6.5 6.0 5.5 5.0 4.5 4.0 ppm
OBn O
N+
BnO OBn
OBn
17
NAME zhh10-29B
EXPNO 13
PROCNO 1
Date_ 20120317
Time 6.36
INSTRUM spect
PROBHD 5 mm DUL 13C-1
PULPROG cosygpqf
TD 2048
SOLVENT CDCl3
NS 1
DS 8
SWH 2913.753 Hz
FIDRES 1.422731 Hz
AQ 0.3514868 sec
RG 71.8
DW 171.600 usec
DE 8.00 usec
TE 295.4 K
D0 0.00000300 sec
D1 1.44634104 sec
D13 0.00000400 sec
D16 0.00020000 sec
IN0 0.00034320 sec
======== CHANNEL f1 ========
NUC1 1H
P0 10.80 usec
P1 10.80 usec
PL1 3.00 dB
SFO1 300.1312917 MHz
====== GRADIENT CHANNEL =====
GPNAM1 SINE.100
GPZ1 10.00 %
P16 1000.00 usec
ND0 1
TD 128
SFO1 300.1313 MHz
FIDRES 22.763695 Hz
SW 9.708 ppm
FnMODE QF
SI 1024
SF 300.1300323 MHz
WDW SINE
SSB 0
LB 0.00 Hz
GB 0
PC 1.40
SI 1024
MC2 QF
SF 300.1300323 MHz
WDW SINE
SSB 0
LB 0.00 Hz
GB 0
NAME zhh10-29B
EXPNO 14
PROCNO 1
Date_ 20120317
Time 6.43
INSTRUM spect
PROBHD 5 mm DUL 13C-1
PULPROG zgpg30
TD 65536
SOLVENT CDCl3
NS 77
DS 4
SWH 17985.611 Hz
FIDRES 0.274439 Hz
AQ 1.8219508 sec
RG 512
DW 27.800 usec
DE 6.50 usec
TE 295.4 K
D1 2.00000000 sec
D11 0.03000000 sec
TD0 1
======== CHANNEL f1 ========
NUC1 13C
P1 12.50 usec
PL1 2.00 dB
SFO1 75.4752953 MHz
======== CHANNEL f2 ========
CPDPRG2 waltz16
NUC2 1H
PCPD2 100.00 usec
PL2 3.00 dB
PL12 22.33 dB
PL13 23.00 dB
SFO2 300.1312005 MHz
SI 32768
SF 75.4677490 MHz
WDW EM
SSB 0
LB 1.00 Hz
GB 0
PC 1.40
137.75 137.42 135.71 128.59 128.51 128.46 128.35 128.14 128.00 127.91 127.84 127.60
76.94 76.34 75.07 74.43 73.68 73.60 71.70 70.02 65.17
OBn O
N+
BnO OBn
OBn
17
140 130 120 110 100 90 80 70 60 50 40 30 20 10 ppm

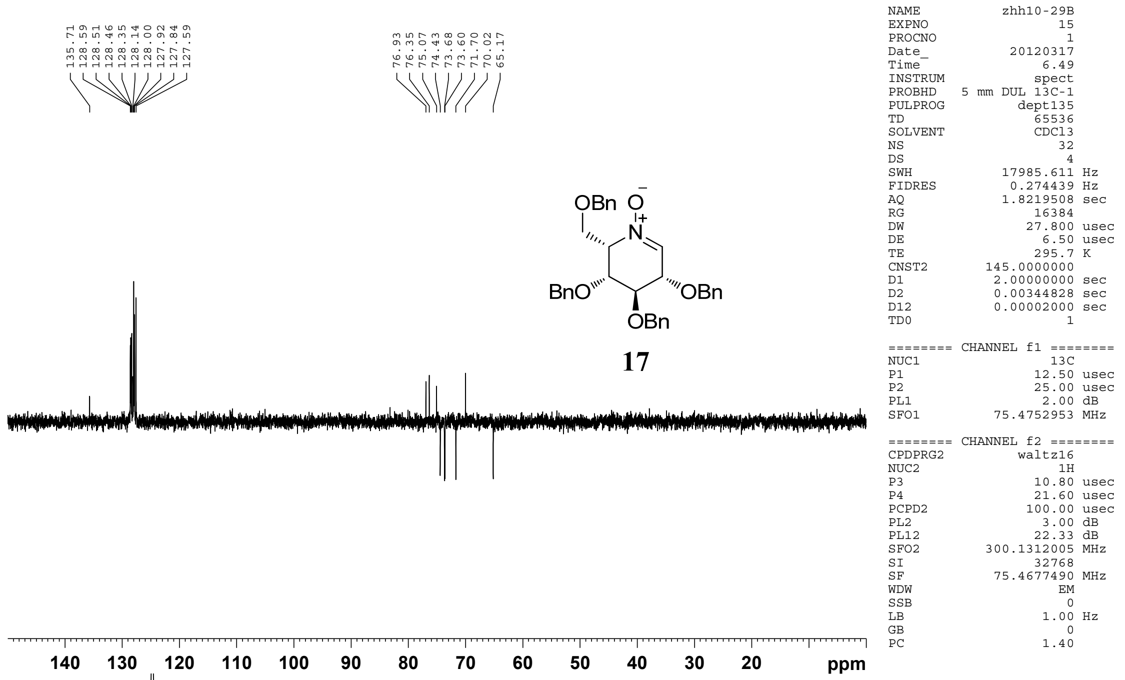
135.71
128.59
128.51
128.46
128.35
128.14
128.00
127.92
127.84
127.59
76.93
76.35
75.07
74.43
73.68
73.60
71.70
70.02
65.17
OBn
O
N+
BnO
OBn
OBn
17
140 130 120 110 100 90 80 70 60 50 40 30 20 ppm
NAME zhh10-29B
EXPNO 15
PROCNO 1
Date_ 20120317
Time 6.49
INSTRUM spect
PROBHD 5 mm DUL 13C-1
PULPROG dept135
TD 65536
SOLVENT CDCl3
NS 32
DS 4
SWH 17985.611 Hz
FIDRES 0.274439 Hz
AQ 1.8219508 sec
RG 16384
DW 27.800 usec
DE 6.50 usec
TE 295.7 K
CNST2 145.0000000
D1 2.00000000 sec
D2 0.00344828 sec
D12 0.00002000 sec
TD0 1
======== CHANNEL f1 ========
NUC1 13C
P1 12.50 usec
P2 25.00 usec
PL1 2.00 dB
SFO1 75.4752953 MHz
======== CHANNEL f2 ========
CPDPRG2 waltz16
NUC2 1H
P3 10.80 usec
P4 21.60 usec
PCPD2 100.00 usec
PL2 3.00 dB
PL12 22.33 dB
SFO2 300.1312005 MHz
SI 32768
SF 75.4677490 MHz
WDW EM
SSB 0
LB 1.00 Hz
GB 0
PC 1.40

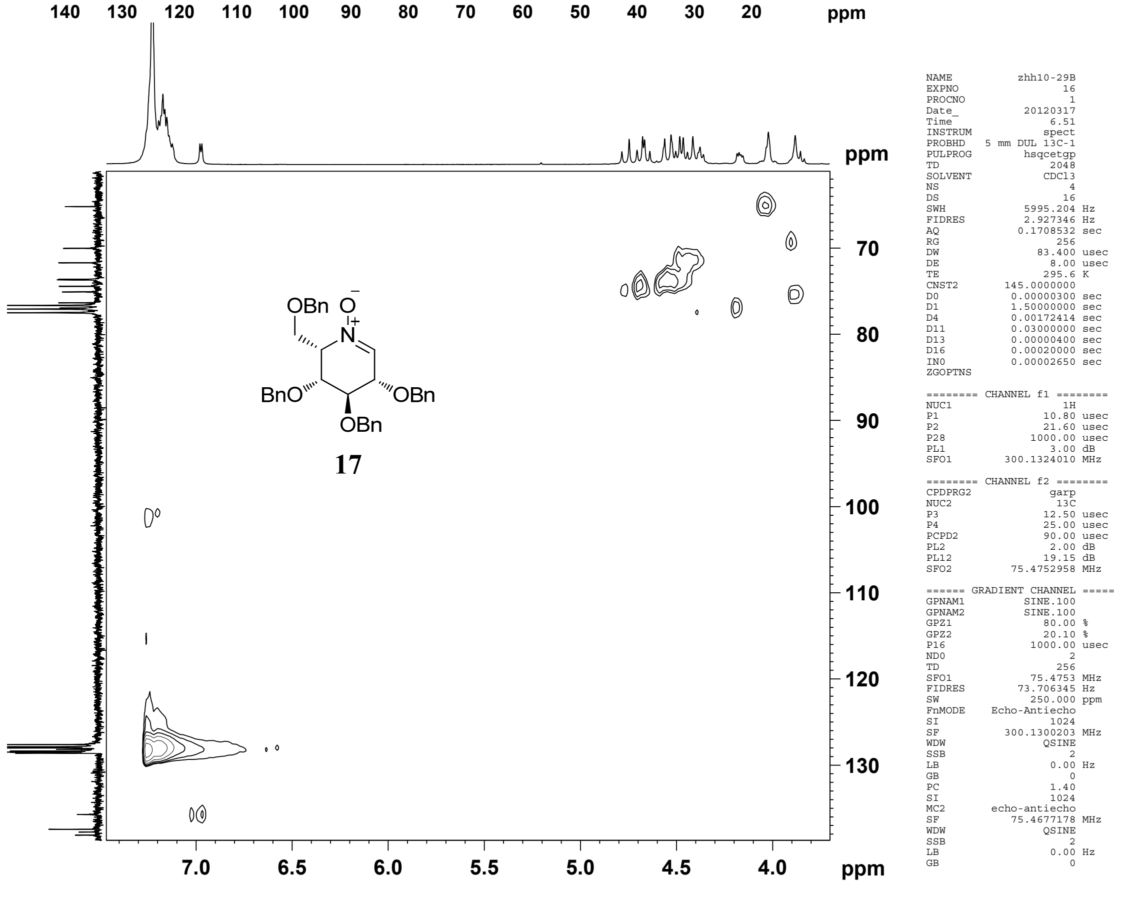
ppm
70
80
90
100
110
120
130
7.0 6.5 6.0 5.5 5.0 4.5 4.0 ppm
OBn
O
N+
BnO
OBn
OBn
17
NAME zhh10-29B
EXPNO 16
PROCNO 1
Date_ 20120317
Time 6.51
INSTRUM spect
PROBHD 5 mm DUL 13C-1
PULPROG hsqcetgp
TD 2048
SOLVENT CDCl3
NS 4
DS 16
SWH 5995.204 Hz
FIDRES 2.927346 Hz
AQ 0.1708532 sec
RG 256
DW 83.400 usec
DE 8.00 usec
TE 295.6 K
CNST2 145.0000000
D0 0.00000300 sec
D1 1.50000000 sec
D4 0.00172414 sec
D11 0.03000000 sec
D13 0.00000400 sec
D16 0.00020000 sec
IN0 0.00002650 sec
ZGOPTNS
======== CHANNEL f1 ========
NUC1 1H
P1 10.80 usec
P2 21.60 usec
P28 1000.00 usec
PL1 3.00 dB
SFO1 300.1324010 MHz
======== CHANNEL f2 ========
CPDPRG2 garp
NUC2 13C
P3 12.50 usec
P4 25.00 usec
PCPD2 90.00 usec
PL2 2.00 dB
PL12 19.15 dB
SFO2 75.4752958 MHz
====== GRADIENT CHANNEL =====
GPNAM1 SINE.100
GPNAM2 SINE.100
GPZ1 80.00 %
GPZ2 20.10 %
P16 1000.00 usec
ND0 2
TD 256
SFO1 75.4753 MHz
FIDRES 73.706345 Hz
SW 250.000 ppm
FnMODE Echo-Antiecho
SI 1024
SF 300.1300203 MHz
WDW QSINE
SSB 2
LB 0.00 Hz
GB 0
PC 1.40
SI 1024
MC2 echo-antiecho
SF 75.4677178 MHz
WDW QSINE
SSB 2
LB 0.00 Hz
GB 0

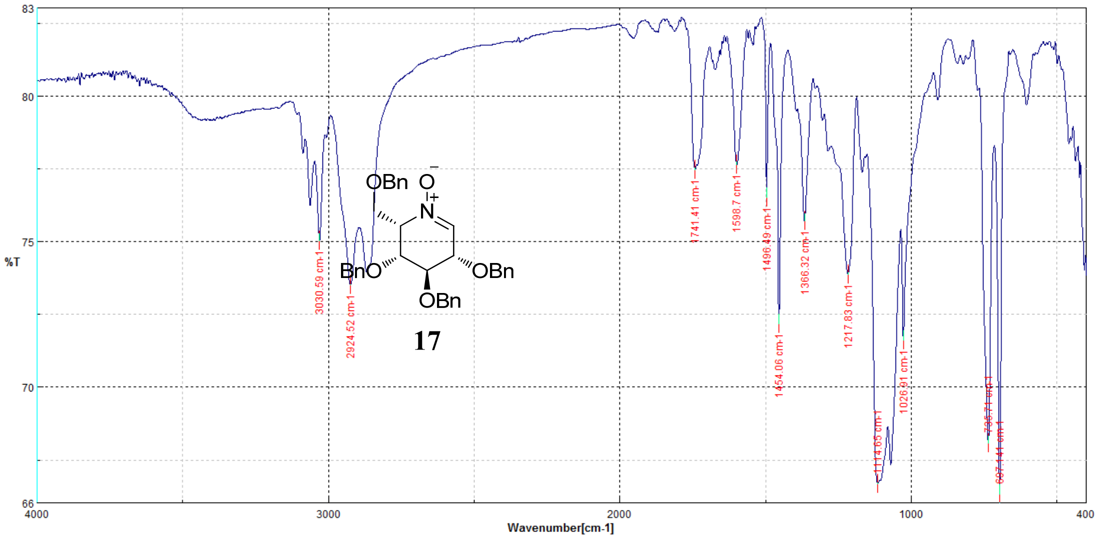
83
80
75
70
66
%T
4000
3000
2000
1000
400
Wavenumber[cm-1]
3030.59 cm-1
2924.52 cm-1
1741.41 cm-1
1598.7 cm-1
1496.49 cm-1
1454.06 cm-1
1366.32 cm-1
1217.83 cm-1
1114.65 cm-1
1026.91 cm-1
735.71 cm-1
697.14 cm-1
OBn
O
N+
BnO
OBn
OBn
17
